# Identification of molecular subtypes and a novel prognostic model of diffuse large B-cell lymphoma based on a metabolism-associated gene signature

**DOI:** 10.1186/s12967-022-03393-9

**Published:** 2022-04-25

**Authors:** Jing He, Ziwei Chen, Qingfeng Xue, Pingping Sun, Yuan Wang, Cindy Zhu, Wenyu Shi

**Affiliations:** 1 Department of Oncology, Affiliated Hospital of Nantong University, 20 Xisi Road, Nantong, 226001 Jiangsu China; 2 Department of Clinical Biobank & Institute of Oncology, Nantong University Affiliated Hospital, Nantong, 226001 China; 3 Department of Cardiology, Affiliated Hospital of Nantong University, 20 Xisi Road, Nantong, 226001 Jiangsu China; 4 Department of Hematology, Affiliated Hospital of Nantong University, 20 Xisi Road, Nantong, 226001 Jiangsu China; 5 Department of Psychology, University of California, Los Angeles (UCLA), Los Angeles, CA 90025 USA

**Keywords:** Diffuse large B-cell lymphoma, Metabolism, Molecular subtype, Prognosis, Immune microenvironment

## Abstract

**Background:**

Diffuse large B cell lymphoma (DLBCL) is the most common lymphoma in adults. Metabolic reprogramming in tumors is closely related to the immune microenvironment. This study aimed to explore the interactions between metabolism-associated genes (MAGs) and DLBCL prognosis and their potential associations with the immune microenvironment.

**Methods:**

Gene expression and clinical data on DLBCL patients were obtained from the GEO database. Metabolism-associated molecular subtypes were identified by consensus clustering. A prognostic risk model containing 14 MAGs was established using Lasso-Cox regression in the GEO training cohort. It was then validated in the GEO internal testing cohort and TCGA external validation cohort. GO, KEGG and GSVA were used to explore the differences in enriched pathways between high- and low-risk groups. ESTIMATE, CIBERSORT, and ssGSEA analyses were used to assess the immune microenvironment. Finally, WGCNA analysis was used to identify two hub genes among the 14 model MAGs, and they were preliminarily verified in our tissue microarray (TMA) using multiple fluorescence immunohistochemistry (mIHC).

**Results:**

Consensus clustering divided DLBCL patients into two metabolic subtypes with significant differences in prognosis and the immune microenvironment. Poor prognosis was associated with an immunosuppressive microenvironment. A prognostic risk model was constructed based on 14 MAGs and it was used to classify the patients into two risk groups; the high-risk group had poorer prognosis and an immunosuppressive microenvironment characterized by low immune score, low immune status, high abundance of immunosuppressive cells, and high expression of immune checkpoints. Cox regression, ROC curve analysis, and a nomogram indicated that the risk model was an independent prognostic factor and had a better prognostic value than the International Prognostic Index (IPI) score. The risk model underwent multiple validations and the verification of the two hub genes in TMA indicated consistent results with the bioinformatics analyses.

**Conclusions:**

The molecular subtypes and a risk model based on MAGs proposed in our study are both promising prognostic classifications in DLBCL, which may provide novel insights for developing accurate targeted cancer therapies.

**Supplementary Information:**

The online version contains supplementary material available at 10.1186/s12967-022-03393-9.

## Background

Diffuse large B-cell lymphoma (DLBCL) is the most common type of non-Hodgkin's lymphoma. About 60% of DLBCL patients experience effective remission after rituximab plus cyclophosphamide, doxorubicin, vincristine, and prednisone (R-CHOP) regimens. However, approximately 30–40% of patients eventually relapse and 10% are primary refractory cases [1]. The International Prognostic Index (IPI), which is widely used to evaluate the prognosis of DLBCL, mainly depends on five traditional clinicopathological features: age, the Eastern Cooperative Oncology Group (ECOG) performance, Ann Arbor stage, lactate dehydrogenase (LDH) level, and extranodal sites, but does not consider the molecular characteristics and microenvironmental differences in lymphoma. Given that DLBCL is a highly heterogeneous tumor, having the same clinicopathological features does not always lead to the same prognosis [2]. Therefore, IPI score is not sufficient to accurately predict the prognosis [3]. It is necessary for us to develop new strategies to identify risk among DLBCL patients more reliably, so as to personalize treatment strategies. Recent studies have shown that risk models based on multi-gene expression are a reliable choice [4–6].

Metabolic reprogramming in tumor cells—notably, aerobic glycolysis, glutamine catabolism, macromolecular synthesis, and redox homeostasis—support the requirements of exponential growth and proliferation [7]. The upregulation of many metabolism-associated genes (MAGs) is driven by the activation of oncogenes. For example, the proto-oncogene c-MYC can activate most glycolytic enzyme genes (principally hexokinase 2(HK2), phosphofructokinase (PFK)-M1, lactate dehydrogenase (LDH)-A and pyruvate kinase M2(PKM2)) to provide fuel for aerobic glycolysis, which subsequently enhances oxidative phosphorylation (OXPHOS). Another oncogene closely related to MAGs is AKT, it directly promotes aerobic glycolysis by upregulating the expression of HK2, PFK1/2 and glucose transporters (GLUT). It can also activate mitochondrial hexokinase (mHK) to promote the coupling of glycolysis and OXPHOS [8, 9]. Therefore, MAGs are considered promising diagnostic markers and potential therapeutic targets. In addition, recent studies have focused on the relationship between metabolism and survival: pan-cancer studies have indicated that tumor subtypes with different MAG expression patterns lead to significantly different survival [10, 11]. Moreover, several risk models based on MAGs have been proposed for breast cancer [6], colorectal cancer [12], gastric cancer [13] and osteosarcoma [14]. However, the value of MAGs in DLBCL subtype identification and prognostic prediction remains unclear.

The tumor microenvironment, as the hotbed of the tumor, has significant immune cell infiltration. In accordance with the complexity of the tumor microenvironment, immune cells recruited to the tumor tissues have dual tumor-promoting and tumor-antagonizing characteristics [15]. The immune microenvironment plays a key role in tumor development and treatment. Studies have shown that metabolic reprogramming is closely related to the tumor immune microenvironment [16]. Metabolites derived from tumor cells can influence the composition and distribution of cells in the immune microenvironment in many ways, ultimately leading to immune dysfunction and tumor progression [17]. For example, metabolic reprogramming can affect the differentiation subtypes and functions of T cells and the polarization and function of macrophages [18]. However, studies on the relationship between MAGs and the immune microenvironment in DLBCL remain limited.

In the present study, we used multiple bioinformatics methods to comprehensively analyze MAGs, identified metabolism-associated molecular subtypes in DLBCL patients, constructed a novel MAG-based risk model evaluating the prognostic value of MAGs in DLBCL, and explored the relationships between MAGs and the immune microenvironment. Finally, two hub genes in the risk model were selected and verified using our own tissue microarray (TMA), and their potential utility as therapeutic targets and diagnostic markers was discussed. Our study may provide new clues on mechanisms and metabolic targets in DLBCL, and it may lay the foundation for accurate immunotherapy that targets metabolic pathways in DLBCL.

## Methods

### Data sources and preprocessing

The GSE10846 Series Matrix File data were downloaded from the National Center for Biotechnology Information (NCBI) Gene Expression Omnibus (GEO) database (the annotation platform was GPL570). The data of 412 DLBCL patients with a complete mRNA expression profile and survival time > 0 was extracted from the GSE10846 dataset. Of the 412 patients in the GSE10846 dataset, 232 patients had undergone R-CHOP treatment, while 180 patients only received CHOP treatment. We randomly divided (4:1 ratio) the 412 cases extracted from the GSE10846 dataset into a training cohort (n = 330) and a testing cohort (n = 82). Additionally, data on DLBC were downloaded from The Cancer Genome Atlas (TCGA) database (https://portal.gdc.cancer.gov/), and 47 DLBCL cases with a complete mRNA expression profile and survival time > 0 were obtained from the TCGA database. We used the TCGA dataset (n = 47) as an external validation cohort to evaluate the predictive efficacy and robustness of the prognosis-associated risk model. Relevant grouping information and clinicopathological features were shown in Table 1.

**Table 1:** Clinicopathological characteristics of the DLBCL cases in GSE10846 and TCGA datasets

Characteristic	GSE10846			TCGA
	All GSE10846 (n = 412)	Training cohort (n = 330)	Testing cohort (n = 82)	Validation cohort (n = 47)
Gender				
Male	222 (53.9%)	173 (52.4%)	49 (59.8%)	26 (55%)
Female	172 (41.7%)	141 (42.7%)	31 (37.8%)	21 (45%)
NA	18 (4.3%)	16 (4.8%)	2 (2.4%)	
Age (year)				
≤ 60	188 (45.6%)	144 (43.6%)	44 (53.7%)	26 (55%)
> 60	224 (54.4%)	186 (56.4%)	38 (46.3%)	21 (45%)
ECOG-PS				
< 2	296 (71.8%)	239 (72.4%)	57 (69.5%)	
≥ 2	93 (22.6%)	75 (22.7%)	18 (22.0%)	
NA	24 (5.8%)	17 (5.2%)	7 (8.5%)	
(COO) Subtypes				
ABC	167 (40.5%)	132 (40.0%)	35 (42.6%)	
GCB	182 (44.2%)	148 (44.8%)	34 (41.4%)	
NA	63 (15.3%)	50 (15.2%)	13 (15.9%)	
LDH level				
Normal	173 (42.0%)	134 (40.6%)	39 (47.6%)	
Elevated	177 (43.0%)	143 (43.3%)	34 (41.5%)	
NA	62 (15.0%)	53 (16.1%)	9 (11.0%)	
Ann Arbor stage				
I–II	188 (45.6%)	147 (44.5%)	41 (50.0%)	
III–IV	217 (52.7%)	176 (53.3%)	41 (50.0%)	
NA	7 (1.7%)	7 (2.1%)		
Extranodal sites				
< 2	297 (72.1%)	236 (71.5%)	61 (74.4%)	
≥ 2	23 (5.6%)	19 (5.8%)	4 (4.9%)	
NA	92 (22.3%)	75 (22.7%)	17 (20.7%)	

The GSE10846 dataset was randomly divided (4:1 ratio) into a training cohort (n = 330) and a testing cohort (n = 82).* ECOG* Eastern Cooperative Oncology Group,* PS* performance status,* LDH* lactate dehydrogenase,* COO* cell-of-origin,* ABC* activated B-cell-like,* GCB* germinal center B-cell-like,* NA* not available

### Identification of metabolism-associated subtypes

MAGs were obtained from the GeneCards database (https://www.genecards.org/). We identified 92 candidate prognosis-related MAGs in GSE10846 by univariate Cox regression. Based on the 92 MAGs, the 412 patients were divided into subgroups with different metabolic expression patterns by consensus clustering using the "ConsensusClusterPlus" R package, and unbiased and unsupervised outcomes were obtained.

### Construction and validation of a MAG-based risk model

The 92 candidate MAGs related to prognosis were selected, and a prognostic model was constructed using least absolute shrinkage and selection operator (LASSO) regression. The risk score formula (based on the expression of each included gene weighted by its LASSO regression coefficient) was constructed using the following format: risk score = ${\sum }_{i=1}^{n}coef*gene expression$. Thereafter, the risk score of each patient was calculated. Using the median risk score as the cutoff, the training cohort was divided into low- and high-risk groups. Survival curves were generated by the Kaplan–Meier method and the two groups were compared using the log-rank test. A time-dependent receiver operating characteristic (ROC) curve analysis was used to study the model prediction accuracy. Cox regression was used to assess the independent prognostic value of the risk score and other clinicopathological features. To provide a reference for predicting the prognosis of DLBCL patients, we used the "rms" R package to construct a nomogram based on the risk score and clinicopathological features, and a calibration plot was used to assess the prognostic ability of the nomogram.

### Immune analyses

The Estimation of Stromal and Immune cells in Malignant Tumor tissues using Expression data (ESTIMATE) method was performed to calculate the stromal score, immune score, ESTIMATE score, and tumor purity. Next, the Cell-type Identification By Estimating Relative Subsets Of RNA Transcripts (CIBERSORT) algorithm was used to analyze the RNA-Seq data of DLBCL patients in order to determine the relative proportions of 22 infiltrating immune cells. Furthermore, to quantify the immune cell infiltration in each sample, single-sample Gene Set Enrichment Analysis (ssGSEA) was used to assess the enrichment of 28 immune cells in the tumor samples. We then calculated the correlations between the risk score and immune regulatory genes, especially immune checkpoints.

### Drug sensitivity analysis and construction of competing endogenouse RNA (ceRNA) network

Based on the Genomics of Drug Sensitivity in Cancer (GDSC) database (https://www.cancerrxgene.org/), which is the largest pharmacogenomics database, we used the "pRRophetic" R package to predict the chemotherapy sensitivity of each tumor sample. The estimated half-maximal inhibitory concentration (IC50) value of each chemotherapy drug was obtained by regression, and the accuracy of regression and prediction was tested by cross-validation with GDSC training set for 10 times. All parameters were selected as default values, including "combat" for removing batch effect and the average value of repeated gene expression. Furthermore, we used FunRich (v3.1.3) and NPInter (v4.0) to construct a ceRNA network based on the model genes.

### Gene Set Variation Analysis (GSVA) and functional enrichment analyses

GSVA is a non-parametric and unsupervised method for evaluating the enrichment of gene sets in relation to mRNA expression data. In this study, gene sets were downloaded from the Molecular Signatures Database (v7.0). Each gene set was comprehensively scored by the GSVA algorithm, and the potential differences in biological functions between the high- and low-risk groups were evaluated. Additionally, to explore the functions of the prognosis-associated MAGs, the "ClusterProfiler" R package was used to annotate the genes with their predicted functions based on Gene Ontology (GO) terms and Kyoto Encyclopedia of Genes and Genomes (KEGG) pathways. GO terms and KEGG pathways with p and q values < 0.05 were deemed statistically significant.

### Weighted Gene Co-expression Network Analysis (WGCNA)

To identify the hub genes among the 14 model genes, we used the WGCNA algorithm. After constructing a weighted gene co-expression network, the gene co-expression modules were identified, and the correlations between gene network and clinical phenotype were explored. The WGCNA-R package was used to construct the co-expression network of all genes in the GSE10846 dataset, and the genes with variance within the first 5000 were identified by the algorithm for subsequent analysis. The soft-threshold β was determined by the function "sft$powerEstimate". The weighted adjacency matrix was transformed into a topological overlap matrix (TOM) to estimate the network connectivity, with hierarchical clustering being used to construct the clustering tree structure of the TOM. Different branches of the clustering tree represented different gene modules, and different colors represented different modules. Tens of thousands of genes were classified into modules based on having similar expression patterns (using their weighted correlation coefficients).

### TMA tissue samples

The DLBCL TMA contained 104 DLBCL tissues and 28 reactive hyperplasia tissues (from cases with the same gender ratio and age range) collected from 2008 to 2015. It was prepared by the Department of Clinical Biobank of the Affiliated Hospital of Nantong University. Clinicopathological data, including gender, age, B symptoms, Ann Arbor stage, hemoglobin (Hb) level, LDH level, IPI score were collected. In addition, X-tile 3.6.1 software was performed to determine the optimal cutoff values for two hub genes expression. This study was a retrospective study, and the informed consent of all patients was obtained before the study. The Ethics Committee of the Affiliated Hospital of Nantong University approved this research.

### Fluorescence-based multiplex immunohistochemistry (mIHC) staining

The DLBCL TMA slides were stained with multiplex fluorescence by using the Opal 7-color Manual IHC Kit (PerkinElmer, MA). After dewaxing by xylene and rehydration by ethanol, slides were heated in a microwave with AR6 Buffer (AR600, AKOYA) and AR9 Buffer (AR900, AKOYA) for antigen retrieval. The slides were incubated with primary antibodies overnight at 4 °C and then incubated with secondary antibody for 10 min at room temperature. At last, we used 4',6-diamidino-2-phenylindole (DAPI; F6057, Sigma) to stain the nuclei and seal the slides. Imaging was achieved using the Vectra 3.0 Automated Quantitative Pathology Imaging System. Tumor and stroma images were captured at ×20 magnification. Finally, the staining was scored by inForm® Cell Analysis software based on the intensity and degree of staining. The degree of staining was compared using the Wilcoxon rank-sum test.

The primary antibodies used in this study were as follows: rabbit anti-PHKA1 (24279-1-AP, Proteintech), rabbit anti-PLTP (ab282456, Abcam), rabbit anti-CD163 (93498, Cell Signaling Technology), rabbit anti-CD68 (76437, Cell Signaling Technology), rabbit anti-CD11B (49420, Cell Signaling Technology), mouse anti-CD66b (ARG66287, Arigobio), rabbit anti-PD-1 (86163, Cell Signaling Technology) and rabbit anti-PD-L1 (13684, Cell Signaling Technology). The secondary antibody was Opal™ polymer HRP Ms + Rb (ARH1001EA, Perkin Elmer).

### Statistical analysis

Survival curves were generated by the Kaplan–Meier method and compared using the log-rank test. Multivariate Cox proportional hazards regression was used to identify independent prognostic factors. Wilcoxon rank-sum test was applied to continuous variables with nonnormal distribution. All statistical analyses were performed in R software (v4.0). All statistical tests were two tailed, and p < 0.05 was considered statistically significant.

## Results

### Identification of prognosis-associated MAGs in DLBCL

The whole study process is depicted in the flow chart in Additional file 1: Figure S1. First, we obtained 958 MAGs from the GeneCards database by using “metabolism” as the search term and setting the relevance score > 5. Second, DLBC mRNA expression data (Fragments Per Kilobase of transcript per Million mapped reads [FPKM]) in the GSE10846 dataset was downloaded, and 877 MAGs were extracted (based on the 958 MAGs identified using GeneCards). To identify the prognosis-associated genes among the 877 MAGs, we used prognostic data on DLBCL patients and obtained 92 prognosis-associated MAGs by univariate Cox regression (p < 0.001; Additional file 2: Figure S2).

### Identifying metabolism-associated molecular subgroups and differences in prognosis and the immune microenvironment between subgroups

**Fig. 1:**
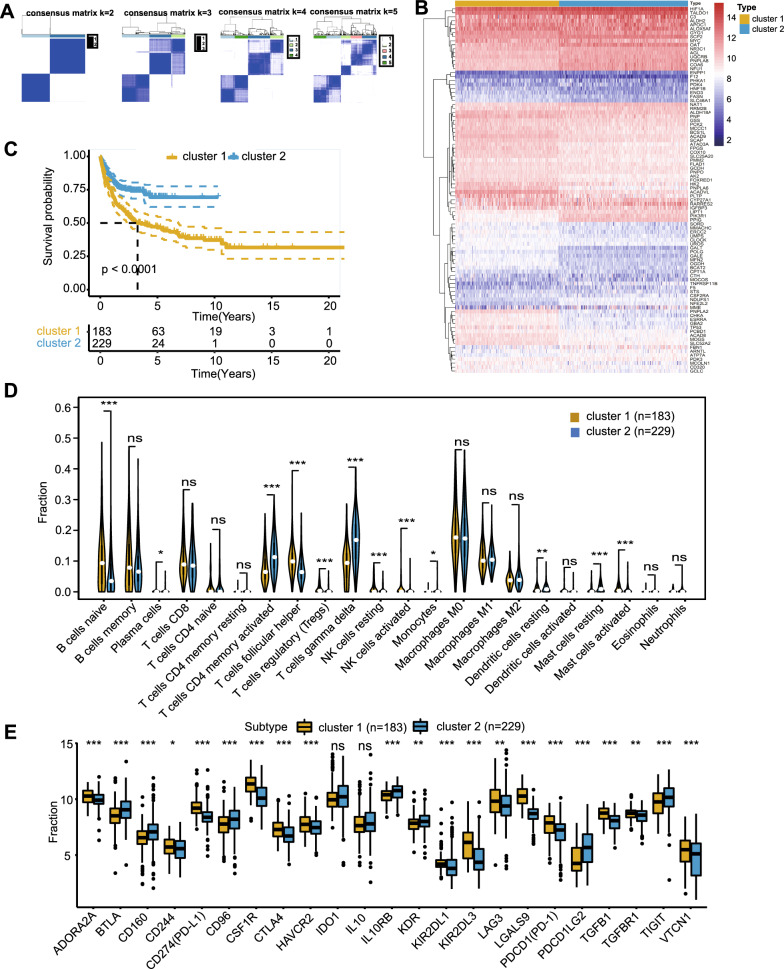
Consensus clustering and the different immune profiles between two clusters. **A **Consensus matrix heatmap indicating that the optimal value for consensus clustering is K = 2. **B **Heatmap visualizing the different expression pattern of the 92 MAGs in the two clusters. **C **Survival curve of the patients in the two clusters. **D **CIBERSORT analysis in the two clusters. **E **The expression of immune checkpoints among two clusters. P values were showed as: ns not significant; *p < 0.05; **p < 0.01; ***p < 0.001

Based on the expression patterns of 92 prognosis-associated MAGs, we used consensus clustering to cluster the 412 patients in the GSE10846 dataset into different metabolism-associated molecular subgroups. By increasing the clustering variable (k) from 2 to 5, we found that consensus clustering was most suitable when k = 2 (Fig. 1A). This indicated that DLBCL patients could be readily divided into two clusters, with 183 patients in cluster 1 and 229 patients in cluster 2. Heatmap visualization showed significant differences in the expression of the 92 MAGs between the two clusters (Fig. 1B). Survival analysis showed that the overall survival in the two clusters was different, with cluster 1 patients having a significantly worse prognosis than cluster 2 patients (follow-up time was 20 years in cluster 1 and 10 years in cluster 2) (Fig. 1C). In addition, the CIBERSORT algorithm was used to evaluate the differences in immune cell infiltration between the two clusters. The abundances of infiltrating immune cells between clusters are shown in Additional file 3: Figure S3, and the quantitative analysis demonstrated that there were significant differences. Cluster 1 had higher infiltration levels of Tregs, NK cells resting and mast cells activated, and lower infiltration levels of T cells gamma delta, T cells CD4 memory activated, monocytes and dendritic cells resting, while cluster 2 showed the opposite trends (Fig. 1D). Analysis of the differences in the expression of immune checkpoints between the two clusters showed that ADORA2A, CD244, CD274, CSF1R, CTLA4, HAVCR2, KIR2DL1, KIR2DL3, LAG3, LGALS9, PDCD1, TGFB1, TGFBR1, and VTCN1 expression levels were significantly higher in cluster 1 than in cluster 2 (Fig. 1E). Higher infiltration of immunosuppressive cells and increased expression of immune checkpoints in the cluster 1 indicated an immunosuppressive tumor microenvironment, which was consistent with the poor prognosis. These findings indicate that the expression of MAGs is related to the prognosis and the immunosuppressive microenvironment in DLBCL patients.

### Functional enrichment analyses of prognosis-associated MAGs and construction of transcriptional regulatory network

We further conducted functional enrichment analyses of the 92 prognosis-associated MAGs. GO analysis indicated that in biological processes (BP), 92 MAGs were mainly enriched in monosaccharide metabolic process, vitamin metabolic process, cofactor metabolic process, cofactor biosynthetic process and small molecule catabolic process. In cell component (CC), 92 MAGs were mainly enriched in cytochrome complex, respiratory chain complex, oxidoreductase complex, mitochondrial inner membrane and mitochondrial matrix. Regarding molecular function (MF), 92 MAGs were mainly enriched in fatty acid derivative binding, acyl-CoA dehydrogenase activity, amide binding, vitamin binding and coenzyme binding (Additional file 4: Figure S4A). Moreover, KEGG analysis revealed similar pathways, including biosynthesis of cofactors, starch and sucrose metabolism, central carbon metabolism in cancer, fatty acid degradation, and biosynthesis of amino acids (Additional file 4: Figure S4B). Combined with the results of GO and KEGG, we found that 92 MAGs were significantly enriched in many key biosynthetic and metabolic pathways, especially the biosynthesis and metabolism of cofactors, which might contribute to the hyperproliferation of tumor cells and dismal outcomes. Furthermore, we used Cytoscape software to construct a protein–protein interaction network of these prognostic MAGs (Additional file 4: Figure S4C).

### Construction and validation of a prognosis-associated risk model composed of 14 MAGs

**Fig. 2:**
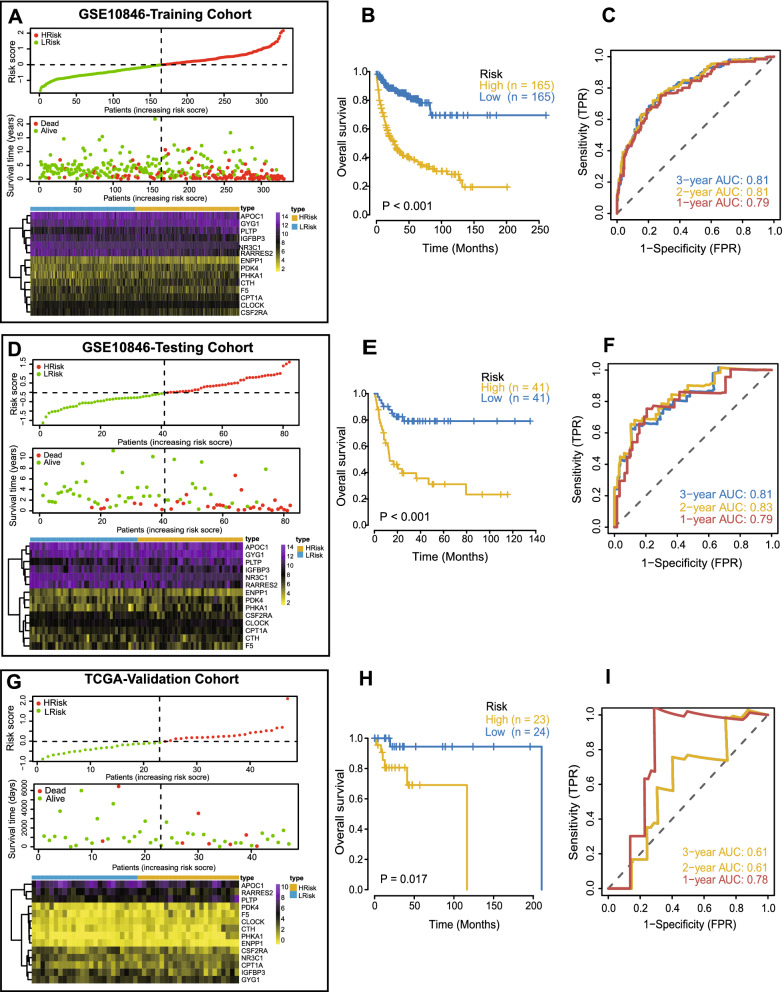
Construction of the risk model in the GSE10846 training cohort and validation of the risk model in the GSE10846 testing cohort and TCGA cohort. **A, D, G **Distribution of the risk score, survival status, and gene expression of 14 MAGs in the GSE10846 training cohort (**A**), GSE10846 testing cohort (**D**) and TCGA cohort (**G**). **B, E, H **Kaplan–Meier curves of OS of patients in the high- and lowrisk groups in the GSE10846 training cohort (**B**), GSE10846 testing cohort (**E**) and TCGA cohort (**H**). **C, F, I **ROC curves for predicting the 1/2/3-year overall survival in the GSE10846 training cohort (**C**), GSE10846 testing cohort (**F**) and TCGA cohort (**I**)

Based on the MAGs identified in univariate Cox regression analyses, we selected 14 MAGs by LASSO regression in order to construct a metabolism-associated prognosis risk model (Additional file 5: Figure S5 and Table 2). The 412 GEO patients were randomly divided (4:1 ratio) into a training cohort (n = 330) and a testing cohort (n = 82). Because of random grouping, the expression patterns of 14 MAGs in the training cohort and the testing cohort were similar. After constructing the model, the risk score of each DLBCL patient in the training cohort was computed based on the following formula: risk score = NR3C1 × (− 0.285969433071478) + IGFBP3 × (− 0.16695536054869) + RARRES2 × (− 0.14291303044122) + F5 × (− 0.0961037965837689) + APOC1 × (− 0.0768487272489624) + CSF2RA × (− 0.056110125649913) + ENPP1 × (− 0.0221430402174049) + GYG1 × 0.0449620982512186 + PHKA1 × 0.0693510636252306 + CPT1A × 0.0752116074259248 + PDK4 × 0.0767229743647787 + CLOCK × 0.0858851468938173 + CTH × 0.108708800807851 + PLTP × 0.16013617077787. Patients were divided into high- and low-risk groups according to the median risk score. The distribution of risk score, survival status, and the expression of the 14 MAGs in the training cohort are depicted in Fig. 2A. Combining the hazard ratio and gene expression heatmap of 14 MAGs, we found that the expression of genes with hazard ratio > 1, such as GYG1, PHKA1, CPT1A, PDK4, CLOCK, CTH, and PLTP, was higher in the high-risk group, while the expression of genes with hazard ratio < 1, such as NR3C1, IGFBP3, RARRES2, F5, APOC1, CSF2RA, and ENPP1, was higher in the low-risk group. Additionally, Kaplan–Meier curves indicated that the DLBCL patients in the high-risk group had significantly worse overall survival (OS) (Fig. 2B). The area under the curve (AUC) values of the ROC curves for 1-, 2-, and 3-year OS were 0.79, 0.81, and 0.81, respectively (Fig. 2C), demonstrating the great predictive performance of the prognosis-associated risk model.

**Table 2:** Features of MAGs in the risk model

No	Gene symbol	Full name	Function	Risk coefficient
1	NR3C1	Nuclear receptor subfamily3 group C member1	Involved in inflammatory responses, cellular proliferation, and differentiation	− 0.28597
2	IGFBP3	Insulin like growth factor binding protein 3	Prolongs the half-life of IGFs and alters their interaction with cell surface receptors	− 0.16696
3	RARRES2	Retinoic acid receptor responder2	As an adipokine and as an antimicrobial protein with activity against bacteria and fungi	− 0.14291
4	F5	Coagulation factor V	Participates with activated coagulation factor X to activate prothrombin to thrombin	− 0.0961
5	APOC1	Apolipoprotein C1	Plays a central role in high density lipoprotein and very low density lipoprotein metabolism	− 0.07685
6	CSF2RA	Colony stimulating factor 2 receptor subunit alpha	Controls the production, differentiation, and function of granulocytes and macrophages	− 0.05611
7	ENPP1	Ectonucleotide Pyrophosphatase/Phosphodiesterase1	Hydrolyzes nucleoside 5′ triphosphates and diadenosine polyphosphates	− 0.02214
8	GYG1	Glycogenin 1	Associated with glycogen storage disease XV	0.044962
9	PHKA1	Phosphorylase kinase regulatory subunit alpha1	Associated with glycogen storage disease type 9D, known as X-linked muscle glycogenosis	0.069351
10	CPT1A	Carnitine palmitoyltransferase 1A	Key enzyme in the carnitine-dependent transport across the mitochondrial inner membrane	0.075212
11	PDK4	Pyruvate dehydrogenase kinase 4	Involved in regulation of glucose metabolism	0.076723
12	CLOCK	Clock circadian regulator	Plays a central role in the regulation of circadian rhythms	0.085885
13	CTH	Cystathionine gamma-lyase	Converts cystathione derived from methionine into cysteine	0.108709
14	PLTP	Phospholipid transfer protein	Involved in regulating the size of HDL particles and cholesterol metabolism	0.160136

Next, we used the GEO testing cohort as an internal validation cohort and the TCGA dataset as an external validation cohort to evaluate the prediction performance and robustness of the prognosis-associated risk model. Using the same formula, we obtained consistent results in the testing cohort, which confirmed the robustness of the risk model. The risk score distribution and gene expression profiles are shown in Fig. 2D. OS was significantly worse in the high-risk group than the low-risk group (Fig. 2E). The AUCs for 1-, 2-, and 3-year OS were 0.79, 0.83 and 0.81, respectively (Fig. 2F). In addition, the TCGA results concurred with the GEO training cohort results. The risk score distribution and gene expression profile are shown in Fig. 2G. Patients with higher risk scores had worse OS (Fig. 2H). The AUCs for 1-, 2-, and 3-year OS were 0.78, 0.61, and 0.61, respectively, indicating that the risk model had a strong prognostic value for DLBCL patients in the TCGA validation cohort (Fig. 2I). In conclusion, these results confirmed that the risk model had a robust and accurate ability to predict OS.

### Clinical correlations and independent prognosis analysis of risk score

**Fig. 3:**
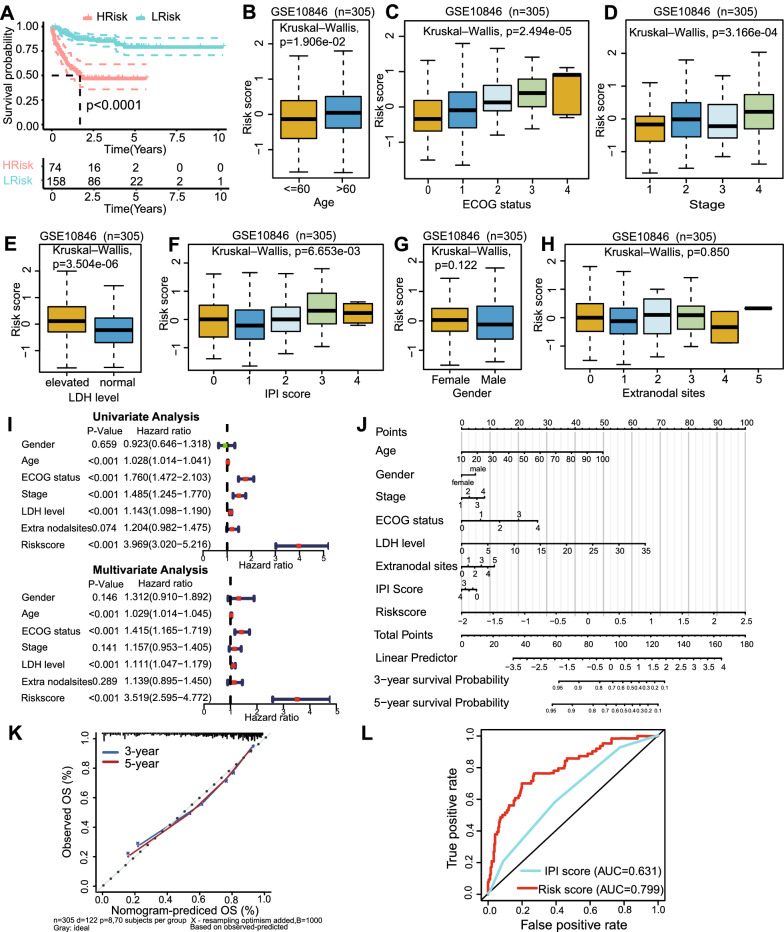
Clinical correlations of risk score and development of the nomogram in the GSE10846 dataset. **A **Survival curve of patients in the high- and low-risk groups in the GSE10846 immunotherapy cohort (232 patients who had received R-CHOP treatment). **B**–**H **Relationships between the risk score and clinicopathological features (including age, ECOG status, stage, LDH level, IPI score, gender and extranodal sites). 305 patients with complete clinicopathological features from the GSE10846 dataset were analyzed. The distance of both ends of boxes represents the interquartile range of values and the thick line represents the median value. **I **Univariate and multivariate analyses revealed the risk score was an independent prognostic factor for DLBCL patients. **J **Nomogram for predicting the 3- and 5-year OS of DLBCL patients. **K **Calibration curves of the nomogram for OS prediction at 3- and 5- year.** L** ROC curves indicating the comparisons of the risk score and the IPI score in predicting 1-year OS

Next, we further validated the clinical value of the risk score. Firstly, we investigated the applicability of the risk score to the immunotherapy cohort. Rituximab (an anti-CD20 monoclonal antibody) plus polychemotherapy (R-CHOP) is the standard of care in DLBCL. Among the 412 patients in GSE10846, only 232 received R-CHOP treatment. We selected these 232 patients as the immunotherapy cohort and compared the survival differences between the high- and low-risk groups in this cohort. The results showed that OS was significantly worse in the high-risk group than the low-risk group (Fig. 3A), indicating that the risk score also had a strong prognostic value for patients who have received immunotherapy. Secondly, to identify whether the risk score was related to clinicopathological features, we compared the risk scores between groups divided based on clinical features, as shown in box plots in Fig. 3B–H. Using Kruskal–Wallis rank sum tests, we found that the risk score significantly differed by age, ECOG status, stage, LDH level, and IPI score (Fig. 3B–F), but it was not related to gender or extranodal sites (Fig. 3G, H). These results indicated that the risk score had good clinical value for classifying DLBCL samples.

In addition, univariate and multivariate Cox regression indicated that risk score was an independent prognostic factor in DLBCL (Fig. 3I). Next, we constructed a prognostic nomogram that integrated the risk score and all significant clinical features. Nomograms can be used to quantitatively predict the prognosis of patients, providing a reference for clinical decision-making. The nomogram demonstrated that the risk score contributed the most to the prognosis, more than the IPI score and other clinical features (such as age, stage, ECOG status, and LDH level) (Fig. 3J). Additionally, the calibration curves for 3- and 5-year OS indicated high consistency between the nomogram predictions and actual observations (Fig. 3K). These findings confirmed that the risk score was a satisfactory prognostic tool for use in DLBCL. Instinctively, we would compare it with the current widely used scoring system, IPI score. Time-dependent ROC curve analysis was further used to determine which scoring system best predicted the OS of DLBCL patients. The AUC of the risk score for predicting 1-year OS (AUC = 0.799) was significantly higher than that of IPI score (AUC = 0.631) (Fig. 3L). This demonstrated that the risk score was superior to the IPI score regarding survival prediction accuracy in DLBCL.

### Relationship between risk score and immune microenvironment

**Fig. 4:**
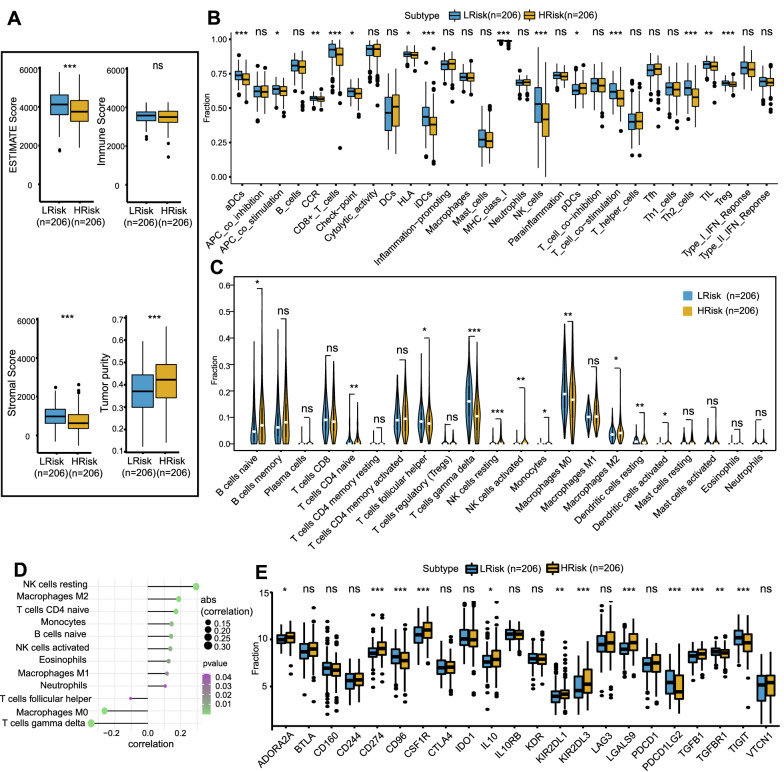
The different immune profiles between the low- and high- risk groups in the GSE10846 dataset. Two risk groups were divided based on the median risk score. **A **ESTIMATE algorithm. **B **ssGSEA analysis. **C **CIBERSORT analysis. **D **Correlation between risk score and immune cell content. **E **Expression variation of immune checkpoint. p values were showed as: ns not significant; *p < 0.05; **p < 0.01; ***p < 0.001

The tumor immune microenvironment significantly affects the therapeutic effect and prognosis of tumor. We assessed the relationship between the risk score and the tumor immune microenvironment in DLBCL by multiple immune analyses. The ESTIMATE results indicated that the patients in the high-risk group had significantly lower stromal and ESTIMATE scores, and higher tumor purity, than those in the low-risk group (Fig. 4A). The ssGSEA was used to assess the immune status of the two groups, which suggesting that the DLBCL patients in the high-risk group had a relatively low immune status (Fig. 4B), which was consistent with the ESTIMATE results. In addition, the results of the CIBERSORT analysis revealed that there were significant differences in most infiltrating immune cells (Additional file 6: Figure S6A). The high-risk group was associated with significantly increased abundances of B cells naive, monocytes, macrophages M2, NK cells resting, NK cells activated, and significantly decreased abundances of T cells CD4 naive, T cells follicular helper, T cells gamma delta, macrophages M0 and dendritic cells resting, while the low-risk group showed the opposite trends (Fig. 4C). Higher abundance of immunosuppressive cells in the high-risk group indicated an immunosuppressive tumor microenvironment, which was consistent with the poor prognosis.

Additionally, we further explored the correlations between the risk score and immune cell content. The results showed that the risk score was positively correlated with macrophages M2, T cells CD4 naive, monocytes, B cells naïve, NK cells resting, and negatively correlated with T cells gamma delta, macrophages M0 and T cells follicular helper (Fig. 4D).

Thereafter, the immune-regulatory genes were further analyzed, and the differences in the expression levels of immune-related chemokines, immunosuppressants, immunostimulants, major histocompatibility complex (MHC) factors, and immune receptors between the high- and low-risk groups are shown in a heatmap (Additional file 6: Figure S6B). As immune cell dysfunction and immunosuppressive microenvironments are characterized by high expression of immune checkpoint-related transcripts, we subsequently focused on the differences in immune checkpoint expression levels and their correlations with the risk score. The results showed that the expression levels of ADORA2A, CD274, CSF1R, IL10, KIR2DL1, KIR2DL3, LGALS9, and TGFB1 were significantly higher in the high-risk group than the low-risk group (Fig. 4E). The correlations between the risk score and the expression of immune checkpoints showed that the risk score was positively correlated with the expression of CD274, CSF1R, KIR2DL1, KIR2DL3, LGALS9, and PVRL2, and negatively correlated with the expression of CD96, PDCD1LG2, TGFBR1, and TIGIT (Additional file 6: Figure S6C). The significantly increased expression of most immune checkpoints in the high-risk group further confirmed that the poor prognosis of high-risk patients was partly related to the immunosuppressive microenvironment. Based on these results, we can reasonably assume that the immunosuppressive microenvironments (characterized by low immune scores, low immune status, high abundance of immunosuppressive cells, and high expression of immune checkpoints) led to poor prognosis in the high-risk group. This is also consistent with our immune analysis of metabolism-associated molecular subtypes. Therefore, the risk model based on 14 MAGs is related to the immunosuppressive microenvironment of DLBCL, and abnormal immune cell infiltration and differential expression of immune checkpoints can be used as prognostic indicators and targets of immunotherapy, which is clinically significant.

### Heterogeneity of drug sensitivity and signaling pathways in high- and low- risk groups

Based on the drug sensitivity data from the GDSC database, we used the "pRRophetic" R package to predict the drug sensitivity of each patient. Gemcitabine, vinblastine, and metformin had lower IC50 values in the high-risk group, while cisplatin and etoposide had higher IC50 values in the high-risk group (Additional file 7: Figure S7A). Furthermore, to explore the discrepancies in signaling pathways between the high- and low-risk groups, GSVA was performed. As shown in Additional file 7: Figure S7B, the differentially enriched signaling pathways between the two groups mainly involved the unfolded protein response, xenobiotic metabolism, KRAS signaling, glycolysis, TGF beta signaling, epithelial–mesenchymal transition, and heme metabolism. The GSVA results suggest that disturbances in these signaling pathways may worsen the prognosis of DLBCL patients in the high-risk group compared to the low-risk group.

### Construction of ceRNA network

To further understand how the 14 MAGs in the risk model regulate mRNA expression by acting as miRNA sponges in DLBCL, we constructed a ceRNA network based on the 14 MAGs. Using FunRich to reverse predict miRNAs based on the 14 MAGs, 57 miRNAs and 130 mRNA-miRNA interactions were identified. Subsequently, using NPInter to reverse predict lncRNAs, 7395 mRNA-miRNA-lncRNA interactions were obtained. Finally, a ceRNA network related to the 14 genes was successfully constructed (Additional file 8: Figure S8). These data may provide clues for identifying the regulatory mechanism of the 14 MAGs in DLBCL.

### Screening for hub genes in the risk model by WGCNA

**Fig. 5:**
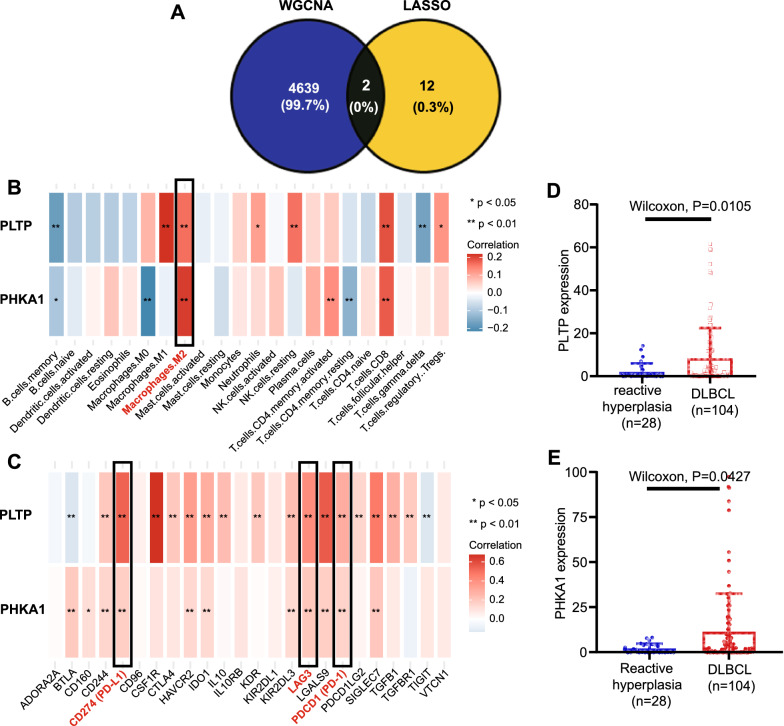
Identification of two hub genes and prediction of their relationship with immune cell content and experimental validation of their expression in the DLBCL TMA cohort. **A **Venn diagram analysis showed that the overlap of WGCNA analysis and LASSO model led to two hub genes being identified: PLTP and PHKA1. **B **Prediction of correlations between hub genes and immune cell content. **C **Prediction of correlations between hub genes and immune checkpoints. **D **Differences in PLTP expression between reactive hyperplasia tissues and DLBCL tissues with mIHC (p value by Wilcoxon rank-sum test). **E **Differences in PHKA1 expression between reactive hyperplasia tissues and DLBCL tissues with mIHC (p value by Wilcoxon rank-sum test)

To identify the most critical genes among the 14 model genes for further experimental verification, the WGCNA algorithm was applied. We constructed a weighted gene co-expression network based on the genes with variance within the first 5000 in the GSE10846 dataset (Additional file 9: Figure S9A). The soft-threshold was set to 1 to build a scale-free network (Additional file 9: Figure S9B). Next, we constructed an adjacency matrix and transformed it into a topological overlapping matrix (TOM). Based on the TOM, three gene modules were identified, namely blue (208), brown (151) and turquoise (4641) modules. Correlation analyses between the modules and clinical trait showed that the turquoise module had the highest correlation (cor = 0.4, p = 2e^−17^) (Additional file 9: Figure S9C). To identify the hub genes among the 14 model genes, we identified the overlapping genes between the turquoise module and the 14 model genes. This led to two hub genes being identified: PLTP and PHKA1 (Fig. 5A). Then, firstly, we explored the correlation between these two hub genes and infiltrating immune cells. We found that both hub genes were positively correlated with the infiltration of M2 macrophages and CD8 T cells (Fig. 5B). Secondly, we further analyzed the correlation between the two hub genes and immune checkpoints. As we expected, we found that PD-1, PD-L1 and LAG3, the common immune checkpoints on T cells, were also positively correlated with both hub genes, which indicated that the highly infiltrated CD8 T cells were dysfunctional (Fig. 5C). Thus, we speculated that these two hub genes were closely related to the immunosuppressive microenvironment and the potential mechanism of these two genes in DLBCL deserved further verification and discussion.

### Experimental verification of hub gene expression and their relationships with prognosis and the immune microenvironment in the DLBCL TMA cohort

**Fig. 6:**
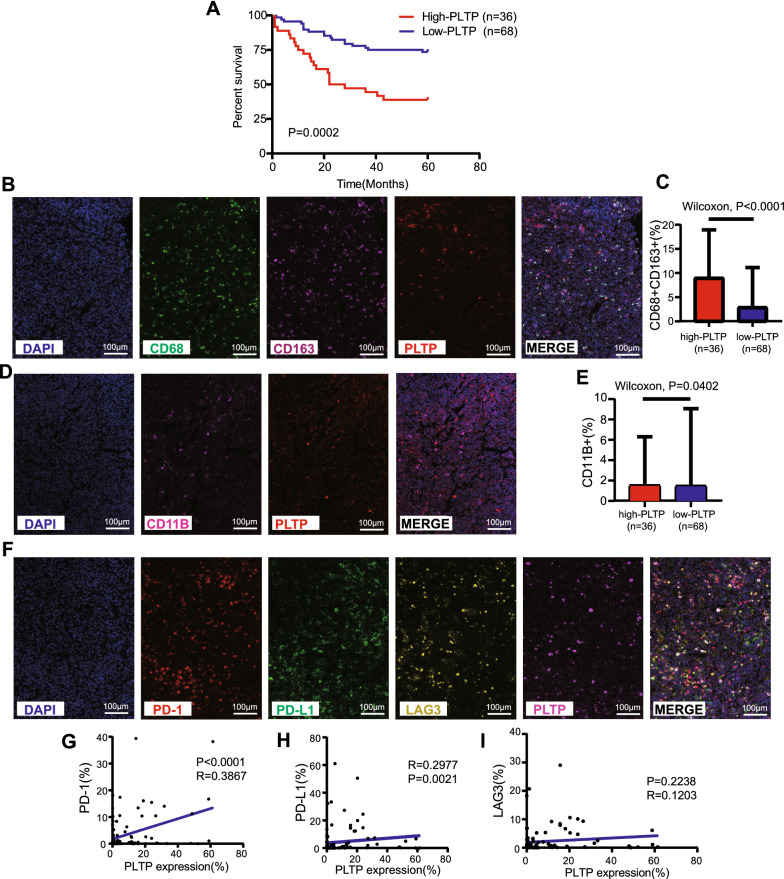
Experimental verification of the relationship between PLTP and prognosis and immune microenvironment in the DLBCL TMA cohort. **A **Kaplan–Meier curve for the PLTP high- and low- expression groups in our TMA cohort. The optimal cutoff point was obtained from X-tile 3.6.1 software. **B **Characterization of cell immunophenotypes with mIHC. A staining panel was developed to visualize DAPI, CD68, CD163 and PLTP simultaneously on the same tissue slide. **C **M2 macrophages content in the PLTP high- and low-expression groups. CD68+CD163+ indicated the content of M2 macrophages (p value by Wilcoxon rank-sum test). **D **Characterization of cell immunophenotypes with mIHC. A staining panel was developed to visualize DAPI, CD11B and PLTP simultaneously on the same tissue slide. **E **TAMs content in the PLTP high- and low- expression groups. CD11B+ indicated the content of TAMs (p value by Wilcoxon rank-sum test). **F **Characterization of cell immunophenotypes with mIHC. A staining panel was developed to visualize DAPI, PD-1, PD-L1, LAG3 and PLTP simultaneously on the same tissue slide. CD274 indicated the content of PD-L1 and PDCD1 indicated the content of PD-1. **G**–**I **Correlations between PLTP expression and immune checkpoints (including PD-1, PD-L1, and LAG3)

**Fig. 7:**
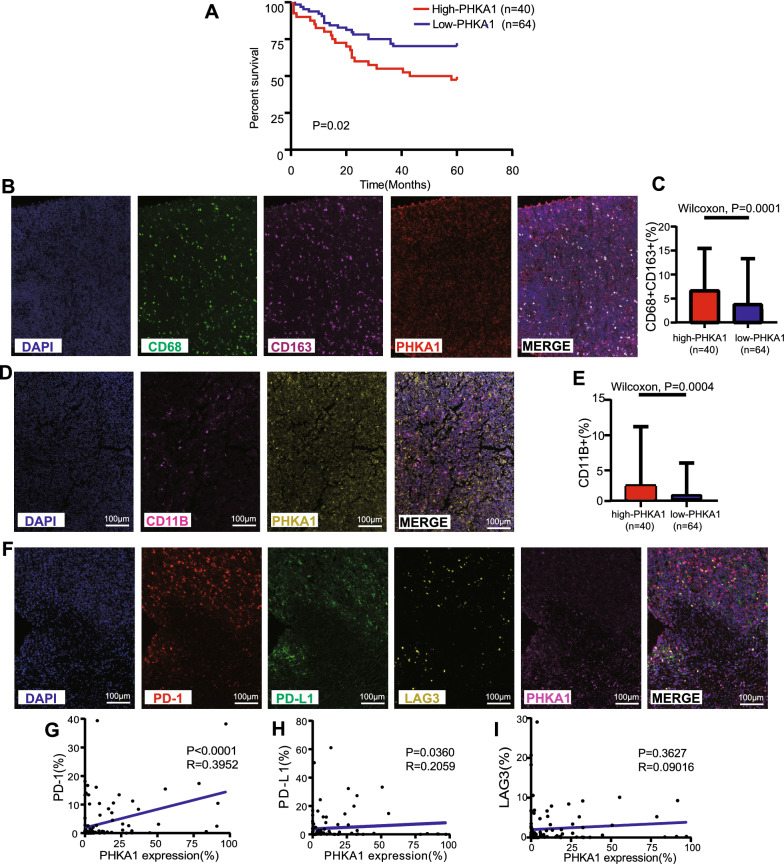
Experimental verification of the relationship between PHKA1 and prognosis and immune microenvironment in the DLBCL TMA cohort. **A **Kaplan–Meier curve for the PHKA1 high- and low-expression groups in our TMA cohort. The optimal cutoff point was obtained from X-tile 3.6.1 software. **B **Characterization of cell immunophenotypes with mIHC. A staining panel was developed to visualize DAPI, CD68, CD163 and PHKA1 simultaneously on the same tissue slide. **C **M2 macrophages content in the PHKA1 highand low- expression groups. CD68+CD163+ indicated the content of M2 macrophages (p value by Wilcoxon rank-sum test). **D **Characterization of cell immunophenotypes with mIHC. A staining panel was developed to visualize DAPI, CD11B and PHKA1 simultaneously on the same tissue slide. **E **TAMs content in the PHKA1 high- and low- expression groups. CD11B+ indicated the content of TAMs (p value by Wilcoxon rank-sum test). **F **Characterization of cell immunophenotypes with mIHC. A staining panel was developed to visualize DAPI, PD-1, PD-L1, LAG3 and PHKA1 simultaneously on the same tissue slide. CD274 indicated the content of PD-L1 and PDCD1 indicated the content of PD-1. **G**–**I **Correlations between PHKA1 expression and immune checkpoints (including PD-1, PD-L1, and LAG3)

Next, we preliminarily verified the two hub genes using mIHC in our own TMA cohort. First, we compared DLBCL tissues with benign reactive hyperplasia tissues, and we found that the expression levels of PLTP and PHKA1 were both significantly increased in DLBCL patients (Fig. 5D, E). Next, X-tile analysis of 5-year OS was performed using the TMA cohort to determine the optimal cutoff values for PLTP and PHKA1 expression. According to the optimal cutoff value for each gene, we divided the 104 DLBCL samples in the TMA cohort into high- and low-expression groups (high-PLTP expression: n = 36, low-PLTP expression: n = 68; and high-PHKA1 expression: n = 40, low-PHKA1 expression: n = 64). Relevant grouping information and clinicopathological features are shown in Tables 3, 4. We then studied the differences in the tumor immune microenvironment between the pairs of groups. Regarding PLTP, the high-expression group had worse prognosis and higher infiltration of M2 macrophages and tumor-associated macrophages (TAMs) compared to the low-expression group (Fig. 6A–E). Moreover, PLTP expression was positively correlated with clinical stage (Table 3) and immune checkpoints PD-1 and PD-L1, but not correlated with LAG3 (Table 3 and Fig. 6F–I). Likewise, regarding PHKA1, the high-expression group had worse prognosis and higher infiltration of M2 macrophages and TAMs compared to the low-expression group (Fig. 7A–E). Moreover, PHKA1 expression was positively correlated with immune checkpoints PD-1 and PD-L1, but not correlated with clinical features and LAG3 (Table 4 and Fig. 7F–I). Finally, univariate and multivariate Cox regression analyses indicated that PLTP and PHKA1 were both independent prognostic factors for patients with DLBCL (Additional file 10: Figure S10). Our verification results confirmed that these two genes were both overexpressed in DLBCL tissues, and their expression levels were closely related to the prognosis and immunosuppressive microenvironment of DLBCL. The conclusions were basically consistent with the results of our bioinformatics analyses.

**Table 3:** Association of PLTP expression levels with clinicopathological characteristics in patients with DLBCL

Characteristic	Total no	Low expression, No. (%)	High expression, No. (%)	Pearson χ^2^	*p*-value
Gender				< 0.0001	0.987
Male	55	36 (65.5)	19 (34.5)		
Female	49	32 (65.3)	17 (34.7)		
Age (year)				0.025	0.874
≤ 60	56	37 (66.1)	19 (33.9)		
> 60	48	31 (64.6)	17 (35.4)		
B symptoms				1.18	0.277
No	84	57 (67.9)	27 (32.1)		
Yes	20	11 (55.0)	9 (45.0)		
Ann Arbor stage				4.007	**0.045** *
I or II	76	54 (71.1)	22 (28.9)		
III or IV	28	14 (50.0)	14 (50.0)		
Hb level (g/L)				2.579	0.108
≥ 120	63	45 (71.4)	18 (28.6)		
< 120	41	23 (56.1)	18 (43.9)		
LDH level (g/L)				0.465	0.495
Normal	51	35 (68.6)	16 (31.4)		
Elevated	53	33 (62.3)	20 (37.7)		
IPI score				0.184	0.668
0–2	55	37 (67.3)	18 (32.7)		
3–5	49	31 (63.3)	18 (36.7)		

^*^P < 0.05. Hb, hemoglobin; LDH, lactate dehydrogenase; IPI, International Prognostic Index

**Table 4:** Association of PHKA1 expression levels with clinicopathological characteristics in patients with DLBCL

Characteristic	Total No	Low Expression, No. (%)	High Expression, No. (%)	Pearson χ^2^	*p-*value
Gender				0.117	0.733
Male	55	33 (60.0)	22 (40.0)		
Female	49	31 (63.3)	18 (36.7)		
Age (year)				3.254	0.071
≤ 60	56	30 (53.6)	26 (46.4)		
> 60	48	34 (70.8)	14 (29.2)		
B symptoms				0.125	0.723
No	84	51 (60.7)	33 (39.3)		
Yes	20	13 (65.0)	7 (35.0)		
Ann Arbor stage				0.313	0.576
I or II	76	48 (63.2)	28 (36.8)		
III or IV	28	16 (57.1)	12 (42.9)		
Hb level (g/L)				0.009	0.924
≥ 120	63	39 (61.9)	24 (38.1)		
< 120	41	25 (61.0)	16 (39.0)		
LDH level (g/L)				0.924	0.336
Normal	51	29 (56.9)	22 (43.1)		
Elevated	53	35 (66.0)	18 (34.0)		
IPI score				0.556	0.456
0–2	55	32 (58.2)	23 (41.8)		
3–5	49	32 (65.3)	17 (34.7)		

Hb, hemoglobin; LDH, lactate dehydrogenase; IPI, International Prognostic Index

## Discussion

The molecular heterogeneity of DLBCL brings great challenges to precision therapy. It is generally accepted that the traditional IPI score cannot adequately predict the prognosis of DLBCL, and developing more reliable strategies for subtype identification and prognostic classification is urgent [3, 19]. In this study, we identified two metabolism-associated molecular subtypes, and there were significant differences in prognosis and the immune microenvironment between these two subtypes. In addition, we developed a prognostic risk model based on 14 MAGs. We found that it was a powerful independent prognostic tool with better predictive performance than the IPI score and was closely related to the immunosuppressive microenvironment. Finally, we identified two hub genes among the model genes, and preliminarily verified them in our own TMA cohort using mIHC. Our results may contribute to the development of accurate immunotherapy for DLBCL that targets metabolic pathways.

Consensus clustering is an unsupervised clustering method that can identify different molecular subtypes according to a gene expression matrix [20]. Using consensus clustering, we identified two metabolism-associated molecular subtypes, which had significant differences in prognosis and the immune microenvironment. Compared to cluster 2, the prognosis of the patients in cluster 1 was poor, accompanied by a high abundance of immunosuppressive cells and a general increase in the expression of immune checkpoints, indicating an immunosuppressive microenvironment. This is consistent with findings regarding other malignancies [6, 13, 14, 21]. As the consensus clustering was based on a MAG expression matrix, we inferred that the expression of MAGs was related to the prognosis and immunosuppressive microenvironment of DLBCL patients.

To further evaluate the prognostic value of the MAGs, we established a 14-gene risk model in the GEO training cohort by univariate Cox regression and LASSO regression. We then constructed a prognostic nomogram that integrated the risk score based on this model and all significant clinical features. The risk score effectively predicted prognosis in the GEO training cohort and was validated in a GEO internal validation cohort and a TCGA external validation cohort. ROC curve analysis confirmed that the risk score was superior to the traditional IPI score. Multiple validation methods indicated the robustness of the risk model, and it is reasonable to believe that this risk model will be broadly applicable for individualized risk management. As previously mentioned, in view of the close relationship between metabolic reprogramming and the tumor immune microenvironment, we performed multiple immune analyses (ESTIMATE, ssGSEA, and CIBERSORT) to explore the differences in the immune landscape between the high- and low- risk groups. As expected, the high-risk group had a poor prognosis and an immunosuppressive microenvironment characterized by low immune score, low immune status, high abundance of immunosuppressive cells, and high expression of immune checkpoints. The low-risk group showed the opposite trend. This is also consistent with our immune analysis of metabolism-associated molecular subtypes. An increased risk score indicates a “cold tumor” [22], with attenuated immunotherapy effectiveness and an immunosuppressive tumor microenvironment caused by metabolic reprogramming, which is consistent with poor prognosis. These conclusions further indicated that MAGs might play important roles in the altered immune response in DLBCL.

Notably, in the two groups with poor prognosis (cluster 1 and the high-risk group), in addition to the increase in the abundance of immunosuppressive cells and the expression of immune checkpoints, there was a significant increase in the infiltration of resting and activated NK cells. This is consistent with the results of previous studies, that is, an increased abundance of activated NK cells is associated with poor prognosis [23]. NK cell dysfunction is common in hematological cancer, and it is related to tumor immune escape [24]. We also found that KIR2DL1 and KIR2DL3 [25], the common immune checkpoints on NK cells, were also significantly overexpressed in cluster 1 and the high-risk group. In the future, immunotherapy that blocks KIR2DL1/KIR2DL3 might reduce the abundance of activated NK cells.

Most of the MAGs in the risk model have been reported to be associated with cancer. To identify the most critical genes, i.e., the hub genes, among the 14 model genes for further experimental verification, we used the WGCNA algorithm to select key genes and then identified the overlapping genes among these genes and the model genes. As a result, we identified two hub genes: PLTP and PHKA1. The potential mechanisms of these two hub genes in DLBCL deserve further discussion.

Phospholipid transfer protein (PLTP) is a widely expressed lipid transfer protein that belongs to the lipopolysaccharide (LPS)-binding/lipid transfer gene family. PLTP can promote the transfer of a series of lipid molecules, including diacylglycerol, phosphatidic acid, sphingomyelin, phosphatidylcholine, phosphatidylglycerol, brain glycosides, and phosphatidylethanolamine. These transport functions play an important role in lipid and lipoprotein metabolism [26, 27]. PLTP is differentially expressed in many kinds of tumors, such as prostate cancer [27], ovarian cancer [28], breast cancer [29], lung cancer [30], gastric cancer [31] and glioma [32]. Such a wide range of cancer types with differential expression of PLTP indicate that PLTP may be an important regulator of some common processes related to tumors.

The phosphorylase kinase regulatory subunit alpha 1 (PHKA1) gene encodes the muscle-type isoform of the PHK alpha subunit [33]. PHKA1 plays a key role in glycogen metabolism [34] and PHKA1 mutations cause glycogen storage disease type 9D, also known as X-linked muscle glycogenosis [35]. However, research on PHKA1 in tumors is still limited. Research has shown that PHKA1, as an important gene related to glycogen metabolism, is related to the metastasis of prostate cancer [36]. In addition, the increased expression of PHKA1 was associated with younger ages of gastrointestinal stromal tumor patients [36].

We further preliminarily validated the two hub genes in our TMA cohort using mIHC, which can quantify immune cells in the tumor microenvironment more objectively than traditional semi-quantitative methods [37]. Our verification results confirmed that the two hub genes were both overexpressed in DLBCL tissues. Thereafter, using X-tile (a valuable tool for outcome-based cutoff optimization) [38] and the 5-year OS of patients, we determined the optimal cutoff value for PLTP and PHKA1 expression. Based on each cutoff value, we subdivided the DLBCL patients into high- and low-expression groups, and further studied the differences in the tumor immune microenvironment between the pairs of groups. We found that the prognosis of the high-expression groups was poorer, accompanied by an immunosuppressive microenvironment characterized by higher abundances of immunosuppressive cells (M2 macrophages and TAMs) and higher expression of immune checkpoints (PD-L1 and PD-1). Finally, univariate and multivariate Cox regression analyses indicated that PLTP and PHKA1 were both independent prognostic factors in DLBCL. These experimental results showed that high expression of the hub genes was closely related to the prognosis and immunosuppressive microenvironment of DLBCL, which was consistent with our bioinformatics analyses, and further verified the stability and accuracy of the risk model.

Studies have shown that metabolic reprogramming is an important feature of immune cell activation. Immune cells have different metabolic characteristics, which affect their immune function [16, 18]. Macrophages, as the main immune-infiltrating cells in solid tumors, can polarize into inflammatory (M1) or immunosuppressive (M2) phenotypes based on external stimuli. M1 macrophages have pro-inflammatory and anti-tumor effects, while M2 macrophages have anti-inflammatory and pro-tumor effects [39]. The metabolic reprogramming of tumors can affect the polarization process of macrophages [40, 41]. For example, hypoxia and lactic acid accumulation can promote the production of immunosuppressive M2 macrophages. The increase in tumor glycolysis produces a large amount of lactic acid, and the accumulation of lactic acid drives macrophages toward the M2 phenotype. M2 macrophages overexpress arginase 1 (ARG1). ARG1 consumes L-arginine, which is necessary for cytotoxic T lymphocytes to exert anti-tumor activity, and produces polyamines with strong immunosuppressive effects [18, 42]. Additionally, hypoxia promotes tumor development by inducing the production of angiogenic factors, mitogenic factors, and cytokines related to tumor metastasis in macrophages [9]. Additionally, macrophages can undergo lipid-based metabolic reprogramming to promote tumor progression via increased membrane cholesterol efflux [43, 44]. Moreover, M2 macrophages up-regulate fatty acid oxidation, mitochondrial respiration, and angiogenesis, thereby promoting tumor progression [9, 45]. Our mIHC results also confirmed that M2 macrophages in DLBCL patients with high metabolic gene expression were significantly increased. Therefore, M2 macrophages may have potential as immunotherapy targets.

Interactions between immune checkpoints and their cognate receptors can deliver inhibitory signals to immune cells leading to their dysfunction and exhaustion, resulting in immunosuppressive microenvironment and tumor progression [46]. Our study showed that the expression of most immune checkpoints significantly increased with increasing risk score, indicating an immunosuppressive microenvironment that was consistent with poor prognosis. Recent studies have shown that immune checkpoints are closely related to metabolism. On the one hand, checkpoint signals can regulate metabolism [18]. For example, PD-L1 in tumor cells can activate the PI3K-Akt-mTOR pathway, stimulate glycolysis, and enhance glucose uptake by the tumor cells [47]. CD155-TIGIT signaling in T cells of human gastric cancer inhibits glucose uptake, lactic acid production, and glycolytic enzyme expression [48]. On the other hand, metabolism also modulate the tumor response to checkpoint blockade immunotherapy. For instance, obesity is recognized to enhance the PD-1 expression, and is associated with better outcome to checkpoint blockade immunotherapy in metastatic melanoma and renal cell carcinoma [46]. Besides, 2-Deoxy-D-glucose (2-DG), a non-metabolizable glucose analog that inhibits normal glucose metabolism, can enhance the efficacy of anti-CTLA-4 immunotherapy by decreasing PD-L1 protein abundance and increasing expression of type-I interferon (IFN) and antigen presentation genes [49]. Moreover, Powell's team showed that a glutamine metabolism inhibitor not only improved the immunosuppressive microenvironment, but also effectively reversed PD-1 inhibitor resistance when combined with a PD-1 inhibitor [50]. Therefore, combining metabolic inhibitors with checkpoint inhibitors is expected to improve the efficacy of checkpoint blockade.

Our research has some unique advantages. In this study, two metabolism-associated DLBCL subtypes were identified, and a risk model based on MAGs was constructed. We used multiple validation methods to evaluate the model: first, we tested the model in a GEO internal testing cohort, then in a TCGA external validation cohort, and finally we identified two hub genes and carried out preliminary verification in our own TMA cohort. Satisfactory results were obtained from the multiple validation methods, confirming the robustness and accuracy of the risk model. In addition, we not only studied the predictive performance of the risk model, but also explored the effect of MAG expression on the tumor immune microenvironment in DLBCL.

## Limitations

Although our findings have potential clinical significance, there are still some limitations. First, the clinical features extracted from the GEO and TCGA databases were limited, as they did not include potential prognostic factors such as smoking and background diseases. Second, this is a retrospective study, and an independent prospective cohort is needed to verify the risk model established in this study. Finally, the value of the two hub genes as potential pharmacological targets needs to be further investigated.

## Conclusions

In this study, we identified two metabolism-associated DLBCL subtypes and constructed a risk model based on MAGs. Our risk model is related to the tumor immune microenvironment and prognosis of DLBCL. This study provides a new 14-gene signature for predicting the prognosis of DLBCL, which may facilitate personalized treatment strategies, and provides an important basis for further study on MAGs and the immune microenvironment.

## Supplementary Information

**Additional file 1: Figure S1.** Flow chart of the data analyzing process. The GSE10846 dataset was randomly divided (4:1 ratio) into a training cohort (n = 330) and a testing cohort (n = 82).

**Additional file 2: Figure S2.** 92 Prognosis-related MAGs by univariate Cox regression.

**Additional file 3: Figure S3.** The proportion of 22 types of immune cells in patients.

**Additional file 4: Figure S4.** Functional analyses based on 92 prognostic MAGs. (A) Lollipop Chart for GO enrichment (BP, biological process; CC, cellular component; MF, molecular function). (B) Barplot graph for KEGG pathways. (C) PPI analysis showing the protein interaction network of MAGs.

**Additional file 5: Figure S5.** LASSO regression analysis revealing the minimum criteria (A, B) and coefficients (C). Blue represented the coefficient greater than 0.1, and yellow represented the coefficient less than 0.1.

**Additional file 6: Figure S6.** The heatmaps of immune cells and immune-regulatory genes expression in the high- and low-risk group. (A) The proportion of 22 types of immune cells in the training cohort patients. (B) Immune-regulatory genes expression in the high- and low-risk group. (C) Correlation between the risk score and immune checkpoints. p values were showed as: ns not significant; *p < 0.05; **p < 0.01; ***p < 0.001.

**Additional file 7: Figure S7.** Prediction of drug sensitivity and signaling pathways in high- and low- risk groups. (A) Sensitivity analysis of common therapeutic drugs in patients of two groups. (B) GSVA analysis for differentially expressed signaling pathways between two groups.

**Additional file 8: Figure S8.** The ceRNA network associated with the model genes. The red dots represent mRNAs, the green dots represent miRNAs and the blue dots represent lncRNAs.

**Additional file 9: Figure S9.** Construction of weighted co-expression network. (A) Clustering dendrogram of 412 patients in the GSE10846 dataset and heatmap of clinical trait (i.e. risk group). According to the median risk score, 412 patients were divided into high- and low- risk groups. In terms of the color in heatmap, white means low-risk group and red means high-risk group. The cluster was based on the genes with variance within the first 5000 in the GSE10846 dataset. (B) Analysis of the scale-free fit index (left) and the mean connectivity (right) for various soft-thresholding power value. (C) Correlation between the modules and clinical trait (i.e. risk group). Blue represents the negative correlation and red represents the positive correlation.

**Additional file 10: Figure S10.** Cox univariate and multivariate regression analysis revealed that PLTP (A) and PHKA1 (B) were both independent prognostic factors for DLBCL patients.

## Data Availability

All the data corresponding to the DLBCL series used in this study are available in GEO (https://www.ncbi.nlm.nih.gov/geo) and TCGA (https://portal.gdc.cancer.gov/), which are public functional genomics data repositories.

## Abbreviations

DLBCL: Diffuse large B cell lymphoma
MAGs: Metabolism-associated genes
GEO: Gene Expression Omnibus
LASSO: Least absolute shrinkage and selection operator
TCGA: The Cancer Genome Atlas
GO: Gene Ontology
KEGG: Kyoto Encyclopedia of Genes and Genomes
GSVA: Gene set variation analysis
ESTIMATE: Estimation of Stromal and Immune cells in Malignant Tumor tissues using Expression data
CIBERSORT: Cell-type Identification By Estimating Relative Subsets Of RNA Transcripts
ssGSEA: Single-sample Gene Set Enrichment Analysis
ceRNA: Competing endogenouse RNA
WGCNA: Weighted gene co-expression network analysis
TMA: Tissue microarray
mIHC: Multiple fluorescence immunohistochemistry
ROC: Receiver operating characteristic
IPI: International Prognostic Index
NCBI: National Center for Biotechnology Information
IC50: Half maximal inhibitory concentration
AUC: Area under the curve
LDH: Lactate dehydrogenase
ECOG: Eastern Cooperative Oncology Group
MHC: Major histocompatibility complex
PD-1: Programmed death-1
PD-L1: Programmed cell death-1 ligand 1
