# Supplementary material for: Identification of molecular subtypes and a novel prognostic model of diffuse large B-cell lymphoma based on a metabolism-associated gene signature
Source: J Transl Med. 2022 Apr 25;20:186. doi: 10.1186/s12967-022-03393-9 (PMC9036805; doi:10.1186/s12967-022-03393-9)
Supplement: Supplementary file 1 — Additional file 1: Figure S1. Flow chart of the data analyzing process. The GSE10846 dataset was randomly divided (4:1 ratio) into a training cohort (n = 330) and a testing cohort (n = 82). [file 12967_2022_3393_MOESM1_ESM.pdf]

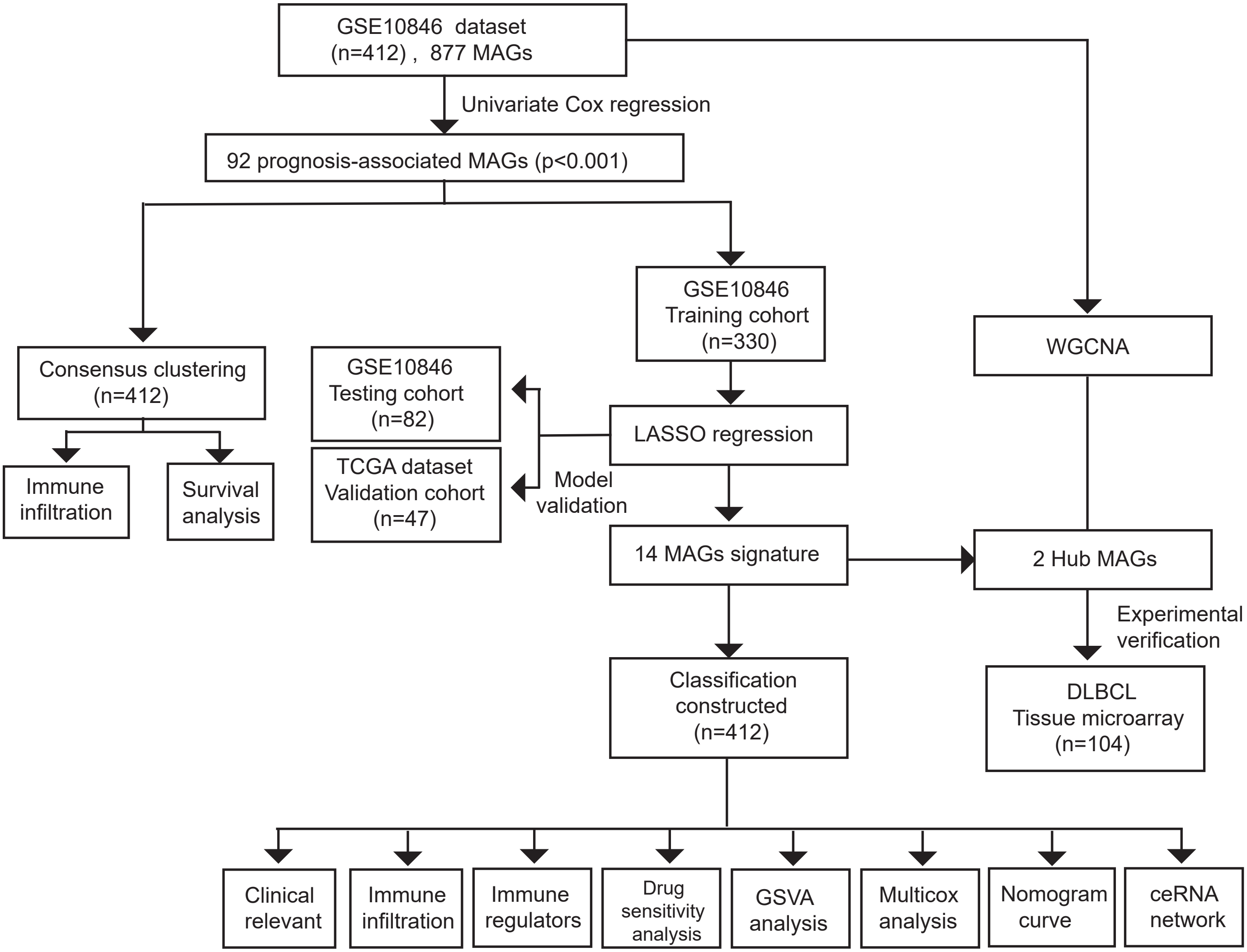

Additional file 1: Figure S1. Flow chart of the data analyzing process. The GSE10846 dataset was randomly divided (4:1 ratio) into a training cohort (n=330) and a testing cohort (n=82).
