# Supplementary figures and images for: Identification of molecular subtypes and a novel prognostic model of diffuse large B-cell lymphoma based on a metabolism-associated gene signature
Source: J Transl Med. 2022 Apr 25;20:186. doi: 10.1186/s12967-022-03393-9 (PMC9036805; doi:10.1186/s12967-022-03393-9)

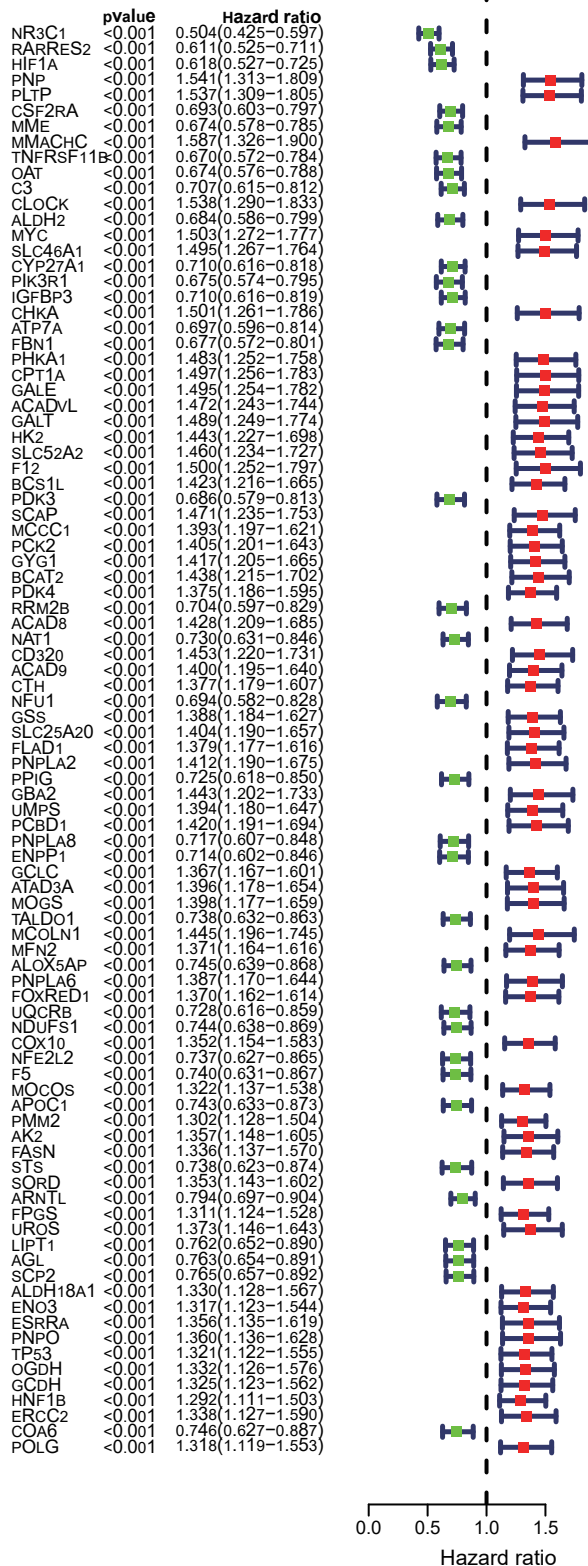

**Additional file 2: Figure S2.** 92 Prognosis-related MAGs by univariate Cox regression.

Supplement: Supplementary file 2 — Additional file 2: Figure S2. 92 Prognosis-related MAGs by univariate Cox regression. [file 12967_2022_3393_MOESM2_ESM.pdf]

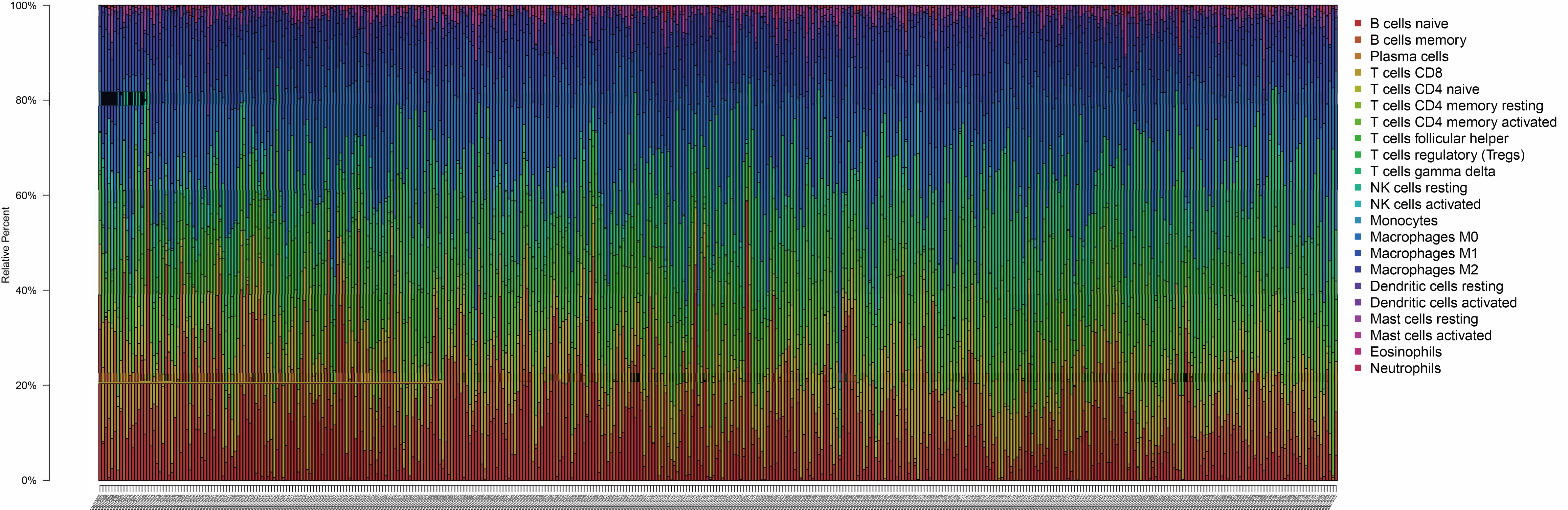

**Additional file 3: Figure S3.** The proportion of 22 types of immune cells in patients.

Supplement: Supplementary file 3 — Additional file 3: Figure S3. The proportion of 22 types of immune cells in patients. [file 12967_2022_3393_MOESM3_ESM.pdf]
