# Supplementary material for: Identification of molecular subtypes and a novel prognostic model of diffuse large B-cell lymphoma based on a metabolism-associated gene signature
Source: J Transl Med. 2022 Apr 25;20:186. doi: 10.1186/s12967-022-03393-9 (PMC9036805; doi:10.1186/s12967-022-03393-9)
Supplement: Supplementary file 5 — Additional file 5: Figure S5. LASSO regression analysis revealing the minimum criteria (A, B) and coefficients (C). Blue represented the coefficient greater than 0.1, and yellow represented the coefficient less than 0.1. [file 12967_2022_3393_MOESM5_ESM.pdf]

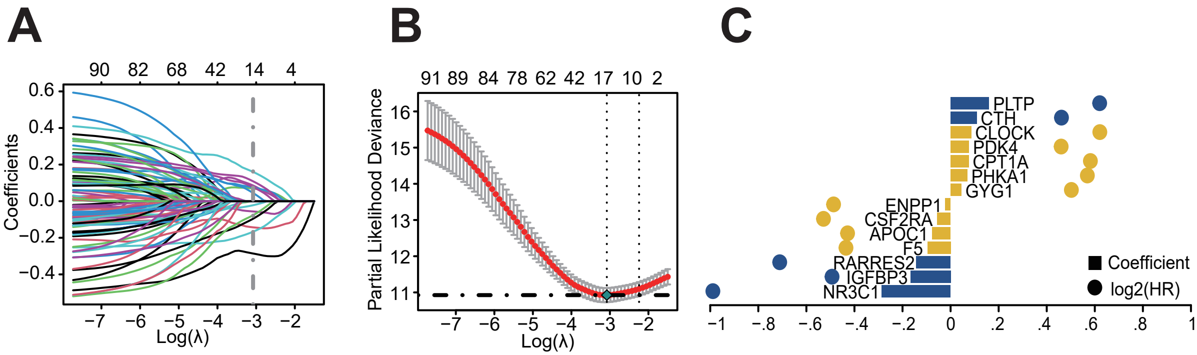

Additional file 5: Figure S5. LASSO regression analysis revealing the minimum criteria **(A, B)** and coefficients **(C).** Blue represented the coefficient greater than 0.1, and yellow represented the coefficient less than 0.1.
