# Supplementary material for: Identification of molecular subtypes and a novel prognostic model of diffuse large B-cell lymphoma based on a metabolism-associated gene signature
Source: J Transl Med. 2022 Apr 25;20:186. doi: 10.1186/s12967-022-03393-9 (PMC9036805; doi:10.1186/s12967-022-03393-9)
Supplement: Supplementary file 6 — Additional file 6: Figure S6. The heatmaps of immune cells and immune-regulatory genes expression in the high- and low-risk group. (A) The proportion of 22 types of immune cells in the training cohort patients. (B) Immune-regulatory genes expression in the high- and low-risk group. (C) Correlation between the risk score and immune checkpoints. p values were showed as: ns not significant; *p < 0.05; **p < 0.01; ***p < 0.001. [file 12967_2022_3393_MOESM6_ESM.pdf]

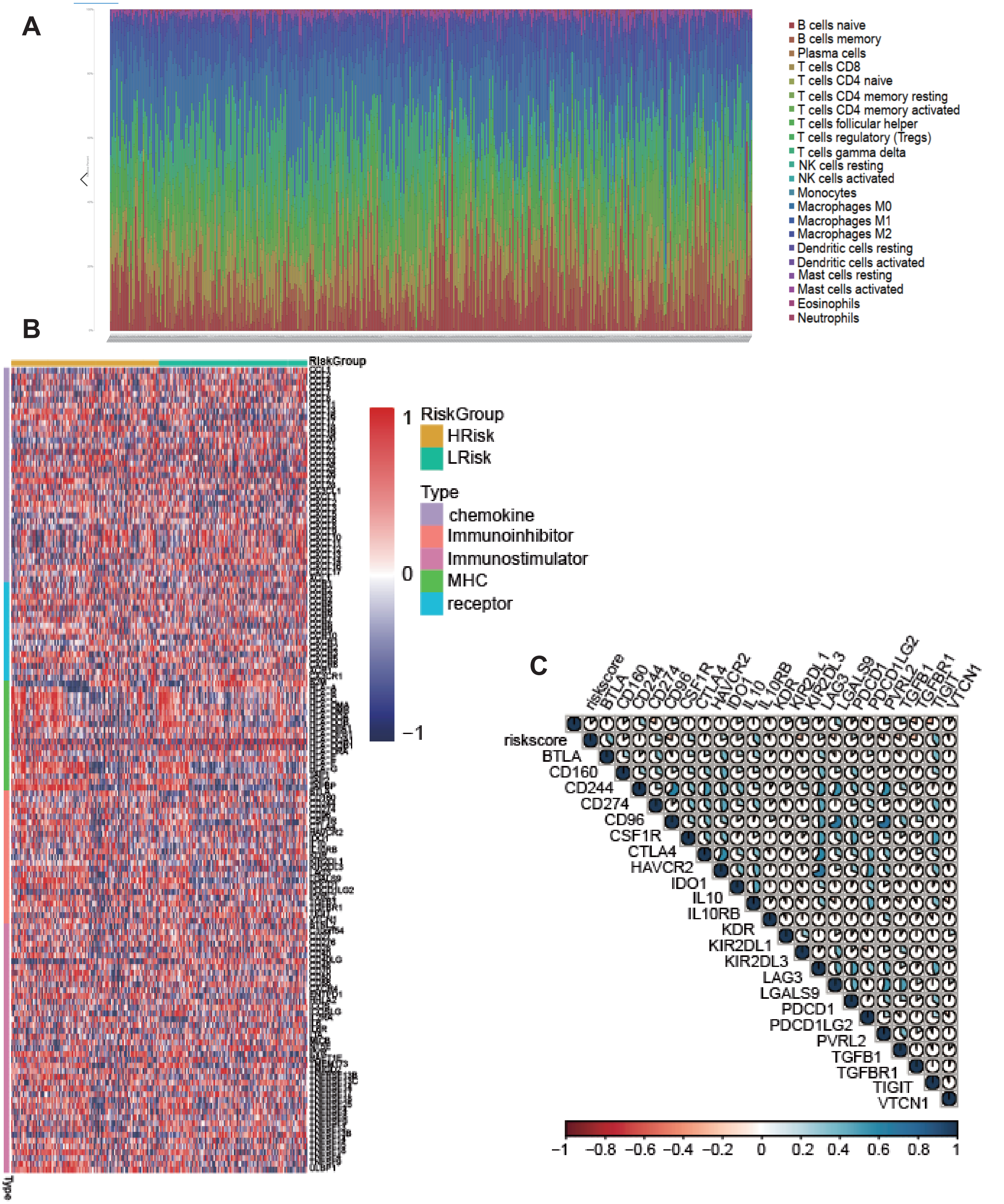

Additional file 6: Figure S6. The heatmaps of immune cells and immune-regulatory genes expression in the high- and low-risk group. **(A)** The proportion of 22 types of immune cells in the training cohort patients. **(B)** Immune-regulatory genes expression in the high- and low-risk group. **(C)** Correlation between the risk score and immune checkpoints. p values were showed as: ns not significant; *p < 0.05; **p < 0.01; ***p < 0.001.
