# Supplementary material for: Identification of molecular subtypes and a novel prognostic model of diffuse large B-cell lymphoma based on a metabolism-associated gene signature
Source: J Transl Med. 2022 Apr 25;20:186. doi: 10.1186/s12967-022-03393-9 (PMC9036805; doi:10.1186/s12967-022-03393-9)
Supplement: Supplementary file 7 — Additional file 7: Figure S7. Prediction of drug sensitivity and signaling pathways in high- and low- risk groups. (A) Sensitivity analysis of common therapeutic drugs in patients of two groups. (B) GSVA analysis for differentially expressed signaling pathways between two groups. [file 12967_2022_3393_MOESM7_ESM.pdf]

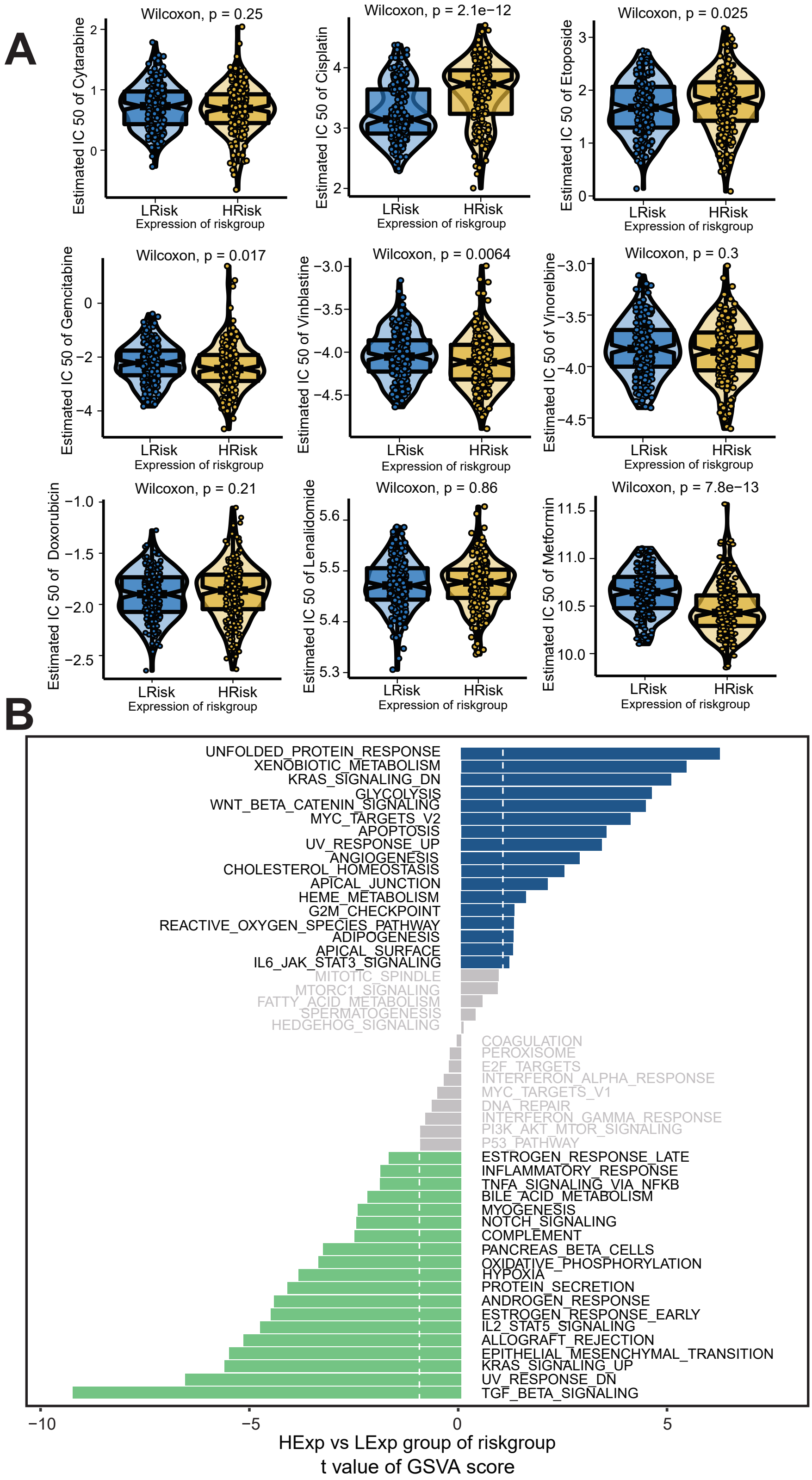

Additional file 7: Figure S7. Prediction of drug sensitivity and signaling pathways in high- and low- risk groups. **(A)** Sensitivity analysis of common therapeutic drugs in patients of two groups. **(B)** GSVA analysis for differentially expressed signaling pathways between two groups.
