# Supplementary material for: Identification of molecular subtypes and a novel prognostic model of diffuse large B-cell lymphoma based on a metabolism-associated gene signature
Source: J Transl Med. 2022 Apr 25;20:186. doi: 10.1186/s12967-022-03393-9 (PMC9036805; doi:10.1186/s12967-022-03393-9)
Supplement: Supplementary file 8 — Additional file 8: Figure S8. The ceRNA network associated with the model genes. The red dots represent mRNAs, the green dots represent miRNAs and the blue dots represent lncRNAs. [file 12967_2022_3393_MOESM8_ESM.pdf]

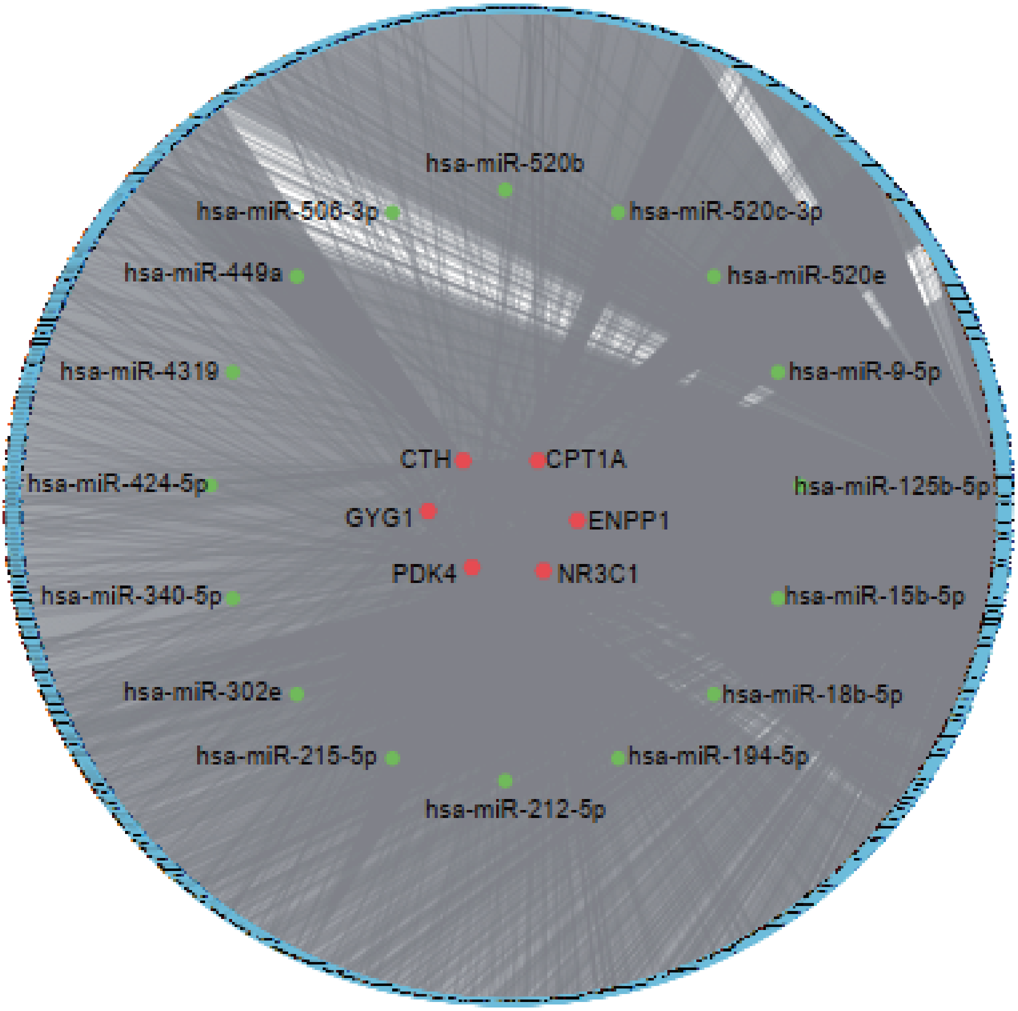

Additional file 8: Figure S8. The ceRNA network associated with the model genes. The red dots represent mRNAs, the green dots represent miRNAs and the blue dots represent lncRNAs.
