# Supplementary material for: Identification of molecular subtypes and a novel prognostic model of diffuse large B-cell lymphoma based on a metabolism-associated gene signature
Source: J Transl Med. 2022 Apr 25;20:186. doi: 10.1186/s12967-022-03393-9 (PMC9036805; doi:10.1186/s12967-022-03393-9)
Supplement: Supplementary file 9 — Additional file 9: Figure S9. Construction of weighted co-expression network. (A) Clustering dendrogram of 412 patients in the GSE10846 dataset and heatmap of clinical trait (i.e. risk group). According to the median risk score, 412 patients were divided into high- and low- risk groups. In terms of the color in heatmap, white means low-risk group and red means high-risk group. The cluster was based on the genes with variance within the first 5000 in the GSE10846 dataset. (B) Analysis of the scale-free fit index (left) and the mean connectivity (right) for various soft-thresholding power value. (C) Correlation between the modules and clinical trait (i.e. risk group). Blue represents the negative correlation and red represents the positive correlation. [file 12967_2022_3393_MOESM9_ESM.pdf]

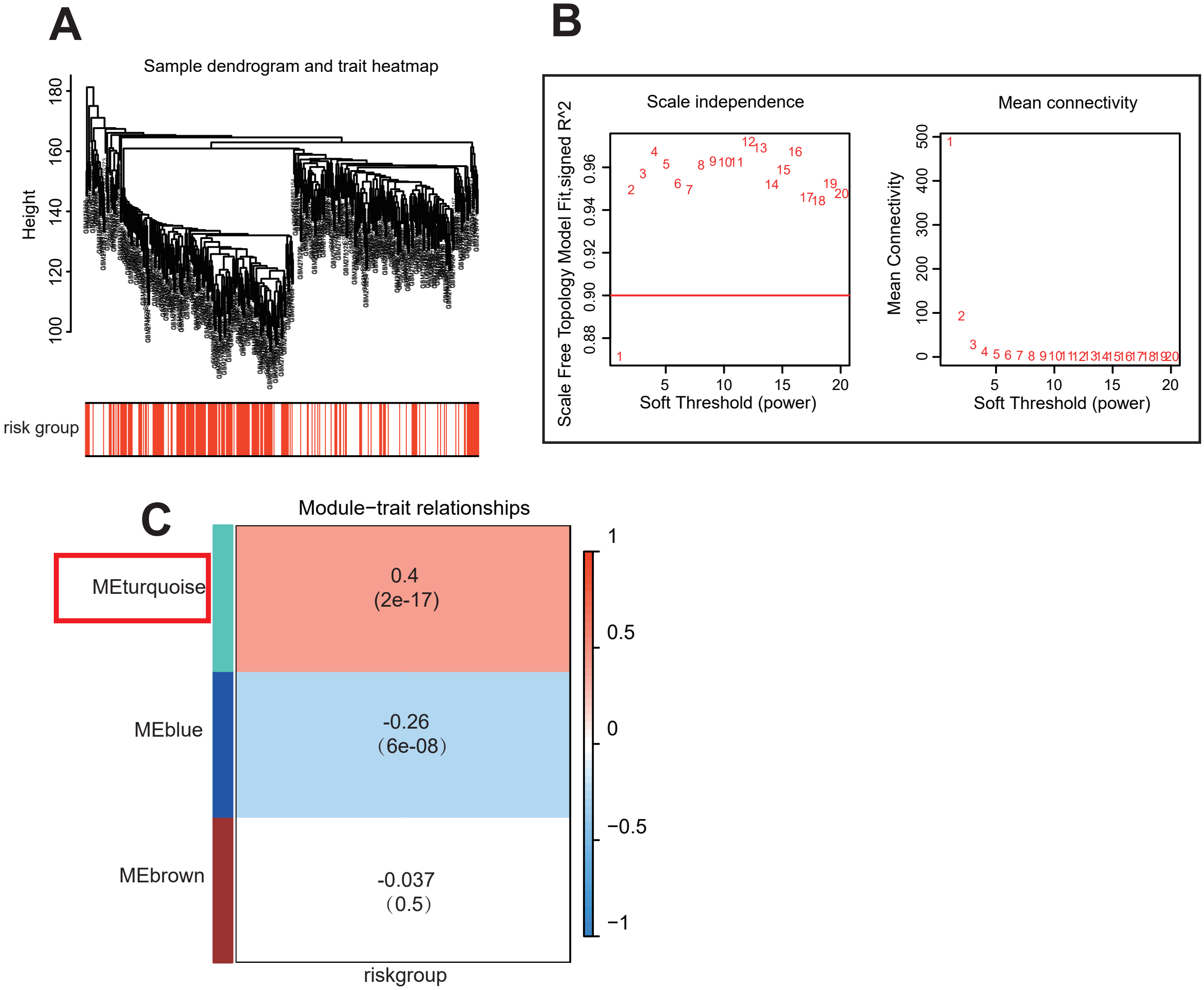

Additional file 9: Figure S9. Construction of weighted co-expression network. **A** Clustering dendrogram of 412 patients in the GSE10846 dataset and heatmap of clinical trait (i.e. risk group). According to the median risk score, 412 patients were divided into high- and low- risk groups. In terms of the color in heatmap, white means low-risk group and red means high-risk group. The cluster was based on the genes with variance within the first 5000 in the GSE10846 dataset. **B** Analysis of the scale-free fit index (left) and the mean connectivity (right) for various soft-thresholding power value. **C** Correlation between the modules and clinical trait (i.e. risk group). Blue represents the negative correlation and red represents the positive correlation.
