# Supplementary material for: Identification of molecular subtypes and a novel prognostic model of diffuse large B-cell lymphoma based on a metabolism-associated gene signature
Source: J Transl Med. 2022 Apr 25;20:186. doi: 10.1186/s12967-022-03393-9 (PMC9036805; doi:10.1186/s12967-022-03393-9)
Supplement: Supplementary file 10 — Additional file 10: Figure S10. Cox univariate and multivariate regression analysis revealed that PLTP (A) and PHKA1 (B) were both independent prognostic factors for DLBCL patients. [file 12967_2022_3393_MOESM10_ESM.pdf]

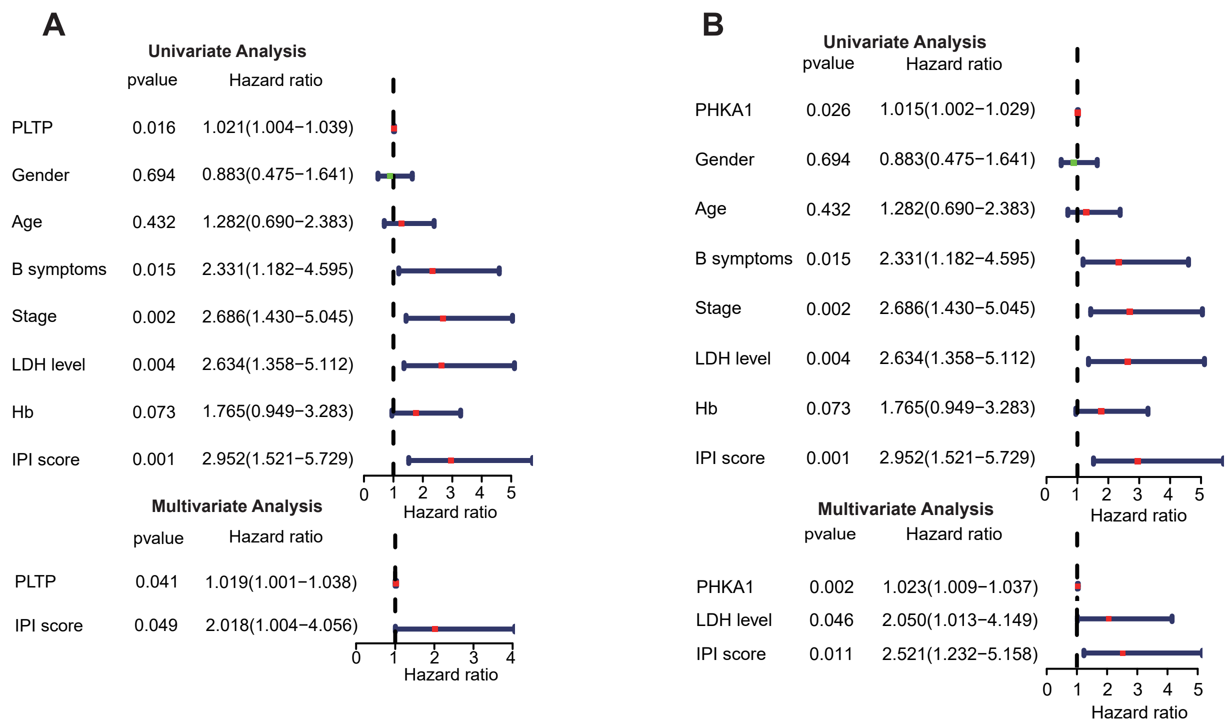


**Additional file 10: Figure S10.**Cox univariate and multivariate regression analysis revealed that PLTP **(A)** and PHKA1 **(B)** were both independent prognostic factors for DLBCL patients.
