# Supplementary material for: Identification of molecular subtypes and a novel prognostic model of diffuse large B-cell lymphoma based on a metabolism-associated gene signature
Source: J Transl Med. 2022 Apr 25;20:186. doi: 10.1186/s12967-022-03393-9 (PMC9036805; doi:10.1186/s12967-022-03393-9)
Supplement: Supplementary file 4 — Additional file 4: Figure S4. Functional analyses based on 92 prognostic MAGs. (A) Lollipop Chart for GO enrichment (BP, biological process; CC, cellular component; MF, molecular function). (B) Barplot graph for KEGG pathways. (C) PPI analysis showing the protein interaction network of MAGs. [file 12967_2022_3393_MOESM4_ESM.pdf]

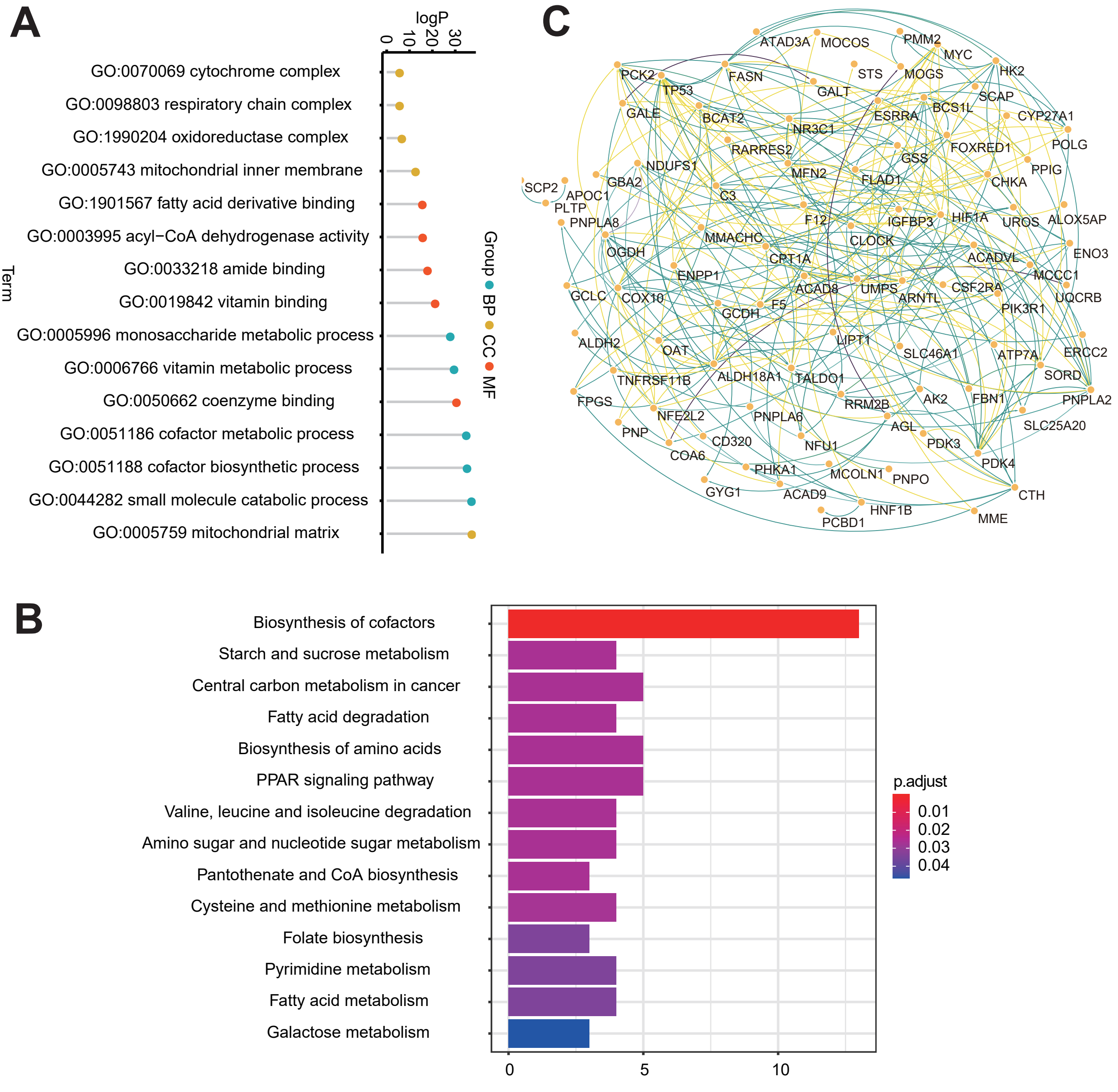

Additional file 4: Figure S4. Functional analyses based on 92 prognostic MAGs. **(A)** Lollipop Chart for GO enrichment (BP, biological process; CC, cellular component; MF, molecular function). **(B)** Barplot graph for KEGG pathways. **(C)** PPI analysis showing the protein interaction network of MAGs.
